# *In situ* biomimetic near-infrared fluorescent protein formed by tumor signature proteins for detecting breast cancer biopsy

**DOI:** 10.1016/j.mtbio.2026.103163

**Published:** 2026-04-27

**Authors:** Jianing Cheng, Taiyu Liu, Xintong Zhu, Chengbin Zhang, Yin Li, Mengxin Li, Dong Song, Jia Li, Feiran Zhang, Shoujun Zhu

**Affiliations:** a State Key Laboratory of Supramolecular Structure and Materials, Center for Supramolecular Chemical Biology, College of Chemistry, Jilin University, Changchun, 130012, PR China; b Joint Laboratory of Opto-Functional Theranostics in Medicine and Chemistry, The First Hospital of Jilin University, Changchun, 130021, PR China; c The School of Pharmaceutical Sciences, Jilin University, 1266 Fujin Road, Changchun, Jilin, 130021, PR China; d Department of Pathology, The First Hospital of Jilin University, Changchun, 130021, PR China; e Department of Breast Surgery, General Surgery Center, The First Hospital of Jilin University, Changchun, 130021, PR China

**Keywords:** Near-infrared fluorescent protein, Tumor signature proteins, Breast cancer biopsy, Three-dimensional histological electrophoresis, Cancer puncture sample detection

## Abstract

Intraoperative rapid diagnosis of breast cancer biopsies currently relies on frozen section pathology or rapid immunohistochemistry. However, the accuracy of these methods is often compromised due to morphological alterations during the freezing process and the limitations of antibody selection inherent to rapid immunohistochemistry (antigen-antibody reactions). To address these issues, we develop a tumor-selective dye that binds *in situ* to tumor signature proteins, forming biomimetic near-infrared fluorescent proteins (NIR-FPs) with much-enhanced brightness. This approach effectively illuminates the tumor region for rapid and accurate identification. The tumor-selective dye inserts into the pockets of tumor signature proteins, then covalently binds with the –SH group of the protein, resulting in a series of NIR-FPs. By combining this with three-dimensional histological electrophoresis, we can select specific molecular weight layers of NIR-FPs (labeled tumor signature proteins) to accurately determine tumor margins. When integrating with the high-throughput 40 × 40 microwell array module, our system demonstrates excellent diagnostic accuracy in processing smaller puncture biopsy specimens. Moreover, the alkyne group in the dye facilitates proteomics analysis of all tumor signature proteins captured by the tumor-selective dye, providing valuable information for identifying new targets for tumor detection.

## Graphical abstract

**Figure:**
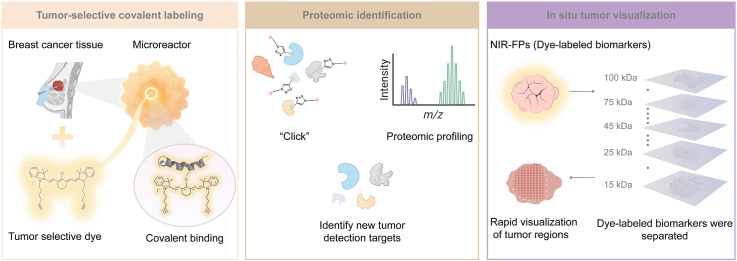

## 1 Introduction

Breast cancer is one of the leading causes of cancer death in women worldwide [[1], [2], [3], [4], [5], [6], [7], [8]]. Accurate and timely diagnosis during biopsy is crucial to formulating an effective treatment plan and improving the prognosis of patients. Despite the progress made in systemic treatment, surgical resection remains the cornerstone of radical treatment for local breast cancer. Among surgical methods, breast preservation surgery (BCS) is increasingly popular because it can maintain a long-term survival rate comparable to mastectomy while preserving breast tissue. The key factor for the success of breast preservation surgery is to completely remove the tumor tissue and ensure that there are no residual tumor cells at the surgical cut edge, because the presence of residual tumor cells at the cut edge will significantly increase the local recurrence risk and the need for reoperation [9,10]. Therefore, timely and accurate evaluation of tumor cutting during surgery is crucial to guide surgical decision-making, improve patient prognosis and reduce the burden of repeated surgery. Among emerging optical approaches, near-infrared (NIR) fluorescence is particularly promising for pathological detection because of its reduced tissue autofluorescence, deeper tissue penetration, and improved signal-to-background performance in biological tissues [11,12].

At present, in clinical practice, intraoperative frozen section analysis (IFSA) and rapid immunohistochemical analysis (IHC) are the main detection methods. IFSA is a standard process that relies on pathologists' morphological assessment of frozen sections. Although informative, this method essentially depends on morphological judgment, which renders its diagnostic accuracy vulnerable to subjective interpretation and subtle histological differences. On the other hand, rapid immunohistochemistry offers another layer of diagnostic precision by targeting specific antigens within the tissue [[13], [14], [15], [16], [17]]. However, this technique also has drawbacks, including the need for additional equipment and trained personnel, limited antibody availability, and potential instability of signal specificity [[18], [19], [20], [21]]. As a result, rapid IHC remains restricted to selected centers and has not been routinely adopted in most hospitals. In addition, the reagents and antibodies used in IHC can be expensive and require careful handling and storage [[22], [23], [24]]. Individual differences of patients can lead to variations in the expression of one or a few tumor signature proteins, affecting the accuracy of intraoperative assessments. To address these challenges, there is an urgent clinical need to establish intraoperative diagnostic strategies that minimize dependence on subjective morphological assessment in IFSA, while also reducing the limitations of antibody-based IHC that arise from restricted marker availability and inter-patient variability. In short, these restrictions highlight the urgent need to combine speed, accuracy and stability, while reducing the need for morphological evaluation and alternative intraoperative diagnostic strategies based on antibody identification.

**Scheme 1:**
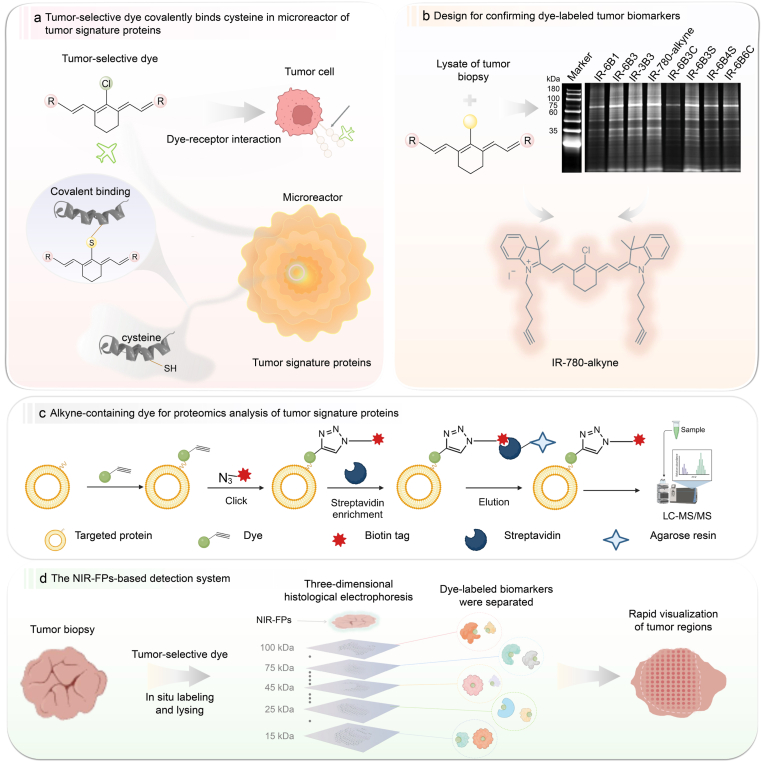
**Schematic workflow of tumor-selective dye *in situ* labeling, proteomic identification, and spatial separation of tumor signature proteins in breast cancer biopsy. (a)** The flow chart of the system utilized tumor-selective dyes as the chromophores, which was covalently bound to the microreactor of tumor signature proteins through nucleophilic substitution reaction. (**b**) SDS-PAGE gel electrophoresis image of protein bands of tumor-selective dyes incubated with lysate of tumor biopsy and design for confirming dye-labeled tumor signature proteins (biomarkers). (**c**) Alkyne-containing dye for proteomics analysis of tumor signature proteins. (**d**) Schematic representation of the NIR-FPs-based system for separating dye-labeled proteins. The tissue section was first placed onto a microwell array for *in situ* lysis and labeling with a tumor-selective dye. Following this step, the lysates were electrophoretically separated by protein mass. The gel layers containing the proteins of interest were then fractionated and imaged to determine the tumor-positive margin. Parts of this Figure were created with BioRender.com.

In this study, we develop a new tumor-selective dye to *in situ* form biomimetic near-infrared fluorescent proteins (NIR-FPs) in tumor biopsy (Scheme 1b). These NIR-FPs are formed through the interaction of the dye with various tumor signature proteins and are engineered for clear imaging of tumor margins. Here, “tumor signature proteins” refers to tumor-associated proteins that reflect the distinct protein composition of tumor tissues relative to normal tissues and may contribute to the preferential labeling behavior of IR-780-alkyne. A series of tumor signature proteins sever as microreactors, facilitating the nucleophilic substitution between dye and proteins (Scheme 1a). By combining this approach with three-dimensional histological electrophoresis to select NIR-FPs with specific molecular weight, their high sensitivity (synergistic multiple tumor signature proteins) and specificity (expression difference between benign and malignant tissues) can enhance the detection of cancerous cells in real-time, eliminating the current dependency on antibodies in rapid immunohistochemistry (Scheme 1d). Moreover, the alkyne group in the dye facilitates proteomics analysis of all tumor signature proteins captured by the dye, clearly defining the formation of biomimetic NIR-FPs (Scheme 1c). We further expressed specific tumor signature proteins and confirmed the covalent binding between the dye and these proteins. The *in situ* targeting of these proteins can simplify the diagnostic workflow, decreasing the reliance on extensive tissue processing and specialized staining procedures. This approach could lead to faster, more accurate intraoperative assessments, thereby improving surgical outcomes and patient care in breast cancer management.

## 2 Results

### 2.1 Chemogenic tumor-selective dyes for constructing biomimetic NIR-FPs

Genetically encoded fluorescent proteins (FPs), such as GFP and RFP, were widely used for long-term tracking of molecules and cells due to their high specificity and intracellular expression [25,26]. However, their application in clinical tissue samples was limited by the need for genetic manipulation and visible-range emission [27]. Inspired by the structural specificity and labeling capacity of FPs, we proposed a novel bionics-driven chemically selective protein labeling strategy from the perspective of bionics to construct NIR-FPs between the dye and series of tumor signature proteins. We speculated that, through a reasonable structural design, Cl-containing dyes could be combined with a large number of proteins with thiol groups, with specific pocket conformations and appropriate sites [[28], [29], [30], [31], [32], [33], [34]]. This combination was expected to develop new tumor-selective dyes that could specifically target a variety of tumor signature proteins, thereby allowing for the quick and automatic distinction of cancer margins.

First of all, we selected eight dyes with strong binding abilities to tumor signature proteins, including IR-6B1, IR-6B3, IR-3B3, IR-780-alkyne, IR-6B3C, IR-6B3S, IR-6B4S and IR-6B6C. These eight dyes had similar absorption and fluorescence spectra (Fig. S1–3). We incubated these dyes with human breast tumor lysate to observe their signature protein labeling abilities. These eight dyes brightly marked multiple proteins in the breast tumor lysate, with similar labeling ability. Next, we compared the dye binding ability to tumor lysates when incubated at 23, 37, and 60^o^C. We found that this bionics-driven chemically selective protein labeling strategy exhibited strong fluorescence properties. The fluorescence intensity was further enhanced with the increase in temperature, which indicated that the increase in temperature may provide additional driving force for the nucleophilic substitution reaction and accelerated the formation of stable NIR-FPs. Through the fluorescent bands exhibited in the gel electrophoresis of dye-labeled tumor lysate, we confirmed that these dyes could mark a variety of signature proteins in tumor samples, demonstrating their ability to selectively label tumors in highly complex biopsy systems.

In subsequent research, we selected the IR-780-alkyne dye, which demonstrated excellent tumor selectivity and photostability (Fig. S4). To further define the optical characteristics of the NIR-FPs-based system, HSA@IR-780-alkyne was employed as a representative model NIR-FP. The model system exhibited an absorption maximum at 787 nm, an emission maximum at 822 nm, and favorable fluorescence stability under ambient light over 12 h (Fig. S5). This enabled us to accurately detect the target protein specifically bound to the dye. This bionics-driven chemically selective protein labeling strategy provided a new method for tumor detection, which was expected to overcome the challenges of uncertain diagnosis caused by tumor variability. In addition, this strategy also addressed the limitations of traditional morphological analysis in tumor margin identification, thereby improving the accuracy and timeliness of diagnosis.

### 2.2 Reaction optimization of tumor signature protein@IR-780-alkyne FPs

To achieve an almost complete response and promote the expansion of production, we took additional steps to optimize the staining conditions for the construction of the “tumor signature protein@IR-780-alkyne fluorescent proteins (FPs).” As the temperature and reaction time increased, the fluorescence intensity of tumor signature protein@IR-780-alkyne FPs gradually increased, with the 60-75 kDa proteins exhibiting a significant increase (Fig. S6). Based on this observation, we defined the 60-75 kDa molecular weight range as the target protein window for subsequent analysis, because proteins within this range exhibited markedly enhanced fluorescence brightness after interacting with the dye, thereby providing better discrimination performance in our system, whereas proteins in the other molecular weight ranges were defined as common proteins.

**Fig. 1:**
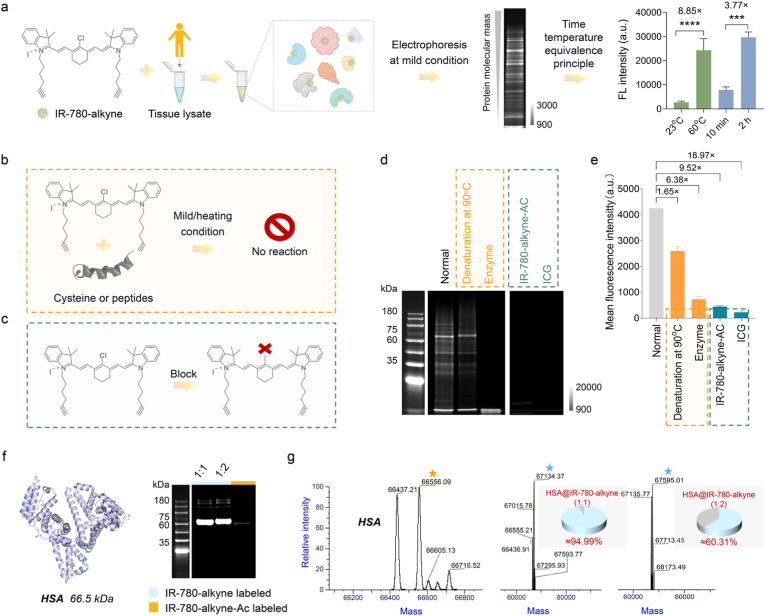
**Binding mechanism of tumor signature protein@IR-780-alkyne FPs. (a)** The diagram of our tumor-selective receptor method. The tumor-selective dye can mark a series of tumor signature proteins. The effect of temperature and time on the fluorescence intensity of the area of interest after the combination of the dye and the tumor tissue lysate was quantified. (**b**) Reaction diagram showing IR-780-alkyne with cysteine or peptides. (**c**) Blocking the reactive chlorine in IR-780-alkyne prevents the labeling reaction. (**d**) SDS-PAGE and fluorescence analysis of breast tumor lysates under different conditions: normal, heated at 90^°^C for 30 min, or digested with trypsin, followed by incubation with IR-780-alkyne at 60^°^C for 2 h. Additional comparisons were made with lysates incubated at 60^°^C for 2 h in the presence of IR-780-alkyne-AC and ICG. (**e**) Quantification of fluorescence intensity from the gel electrophoresis in (d). (**f**) SDS-PAGE gel electrophoresis image of HSA@IR-780-alkyne (1:1), HSA@IR-780-alkyne (1:2), and HSA@IR-780-alkyne-AC. (**g**) High-resolution mass spectrometry of HSA, HSA@IR-780-alkyne (1:1), and HSA@IR-780-alkyne (1:2). Parts of schemes were created with BioRender.com.

We hypothesized that with the increase of reaction temperature and reaction time, the effect of the microreactor would be enhanced, thereby leading to stronger binding between the protein and dye. Compared with the traditional multicolor immunofluorescence staining method, the chemically selective protein labeling strategy can comprehensively label a series of tumor signature proteins. It was not only simpler and faster, but also allowed protein-labeling under mild physiological conditions without additional catalysts. We also quantified the effects of reaction temperature and reaction time on dye-labeled tumor signature proteins. The results confirmed that reaction temperature had a greater impact on the fluorescence intensity of the target proteins@IR-780-alkyne FPs than reaction time (Fig. 1a). This finding further verified that increasing the temperature could improve the labeling efficiency and fluorescence properties of dyes. Collectively, this bionics-driven chemically selective protein labeling strategy not only enhanced the performance of fluorescent dyes but also provided a more accurate and faster solution for tumor edge detection and imaging-guided surgery.

### 2.3 Binding mechanism of tumor signature protein@IR-780-alkyne FPs

We conducted a series of experimental verifications to obtain a better understanding of the reaction mechanism between IR-780-alkyne and breast tumor lysate. First, we digested the protein from the cleavage of the breast tumor with trypsin, which destroyed its protein hydrophobic cavity structure (microreactor) and decomposed it into smaller peptide segments. Through gel electrophoresis analysis, we found that even under heating conditions, these peptide chains did not react with IR-780-alkyne. This result indicated that the binding of dyes exhibited stronger dependence on protein structures than on specific sequences of peptide chains.

To further verify this hypothesis, we heated the breast tumor lysate at 90^°^C for 30 min to induce partial protein denaturation, and then reacted it with IR-780-alkyne under the same conditions. The results revealed that although the fluorescence intensity decreased, IR-780-alkyne could still bind to the proteins (Fig. 1b and Fig. S7a). We speculated that although the protein underwent partial denaturation at high temperatures, its thermal stability and pocket conformation structure were not completely destroyed. Therefore, we inferred that IR-780-alkyne primarily combined with the pocket conformation of breast tumor signature proteins, rather than relying on specific sequence interactions of peptide chains. As long as the pocket conformation was relatively conserved, the dye could still bind effectively even with moderate structural variations of the protein.

Based on the mechanism of interaction between dyes and proteins, we inferred that IR-780-alkyne was inserted into the protein structure through supramolecular interactions, and then formed covalent bonds between chlorine (-Cl) in the dye and the site-specific thiol (-SH) groups of the protein. To confirm that the fluorescence signal of IR-780-alkyne originated from covalent binding instead of non-specific binding, we constructed a derivative of IR-780-alkyne, IR-780-alkyne-AC without C-Cl group (Fig. 1c). This derivative exhibited similar supramolecular interactions as IR-780-alkyne but did not form covalent bonds with protein molecules. At the same time, we also analyzed ICG dye (Fig. 1d and e) and found that they did not covalently combine with tumor lysate, but instead exhibited non-specific accumulation (Fig. S7b and Fig. S8a).

To further optimize the reaction efficiency between dyes and tumor signature proteins, we tested reaction concentration on the optical properties of IR-780-alkyne. We confirmed through electrophoresis that IR-780-alkyne dye bound to breast tumor signature proteins through covalent binding. When the reaction concentration of IR-780-alkyne exceeded 20 μM, that is, when the ratio of protein to dye exceeded 1:2, IR-780-alkyne remained too much, indicating that the dye and protein had not fully reacted (Fig. S8b). We further used human serum albumin (HSA) to study the binding mechanism through LC-HRMS (Fig. 1f). HSA molecule covalently bound two IR-780-alkyne molecules when incubated at 1:1 reaction ratio, but still retained a large proportion of unbound HSA. When the reaction ratio changed from 1:1 to 1:2, all HSA molecules were bound by IR-780-alkyne dye. Therefore, the final best reaction ratio was 1:2 (Fig. 1g and Fig. S9). We speculated that there might be two independent binding sites for IR-780-alkyne dye on HSA. These binding results on the model protein prompted us to increase the dye-to-protein ratio to achieve more thorough binding of the tumor signature proteins.

### 2.4 Confirmation of tumor signature proteins captured by IR-780-alkyne

**Fig. 2:**
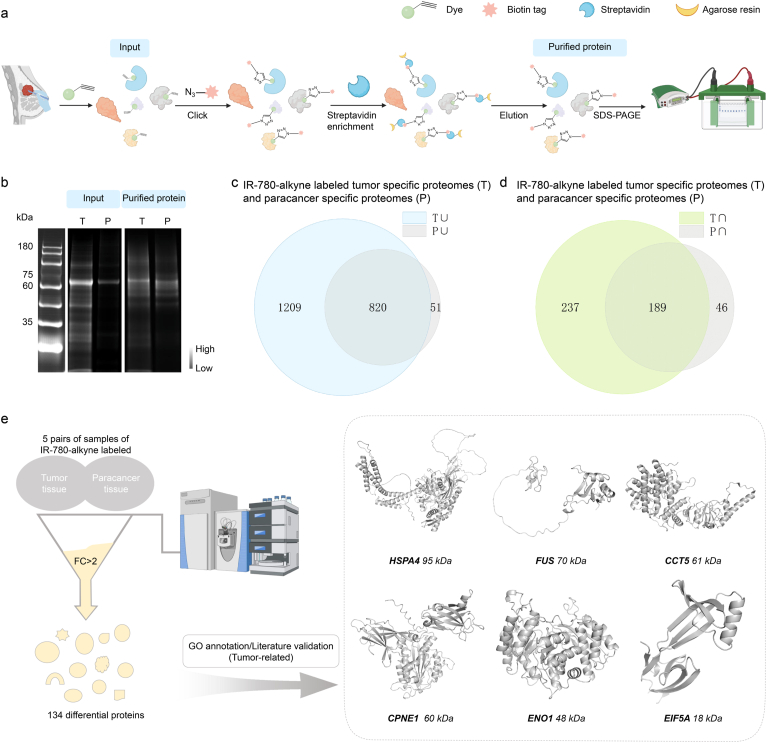
**Confirmation of tumor signature proteins captured by IR-780-alkyne.** (**a**) Purification process of tumor signature protein captured by IR-780-alkyne. (**b**) SDS-PAGE gel electrophoresis image of R0445 and IR-780-alkyne after incubation and purified protein. (**c**) Venn diagram showing the intersection between the union of proteins captured by IR-780-alkyne in five tumor samples (T∪) and the union of proteins captured in five paracancerous samples (P∪). (**d**) Venn diagram showing the intersection between the shared proteins captured by IR-780-alkyne in five tumor samples (T∩) and those in five paracancerous samples (P∩). (**e**) Proteomic analysis of five paired tumor and paracancerous tissues identified 134 differentially enriched proteins. GO annotation and literature validation revealed that HSPA4, FUS, CCT5, CPNE1, ENO1, and EIF5A are highly associated with tumor progression. Experimentally determined structures were generated from the RCSB Protein Data Bank. For proteins without available PDB entries, predicted models were taken from the AlphaFold Protein Structure Database. Parts of schemes were created with BioRender.com.

The alkyne group in the dye promoted the proteomic analysis of all tumor signature proteins captured by the tumor-selective dye. Our subsequent goal was to identify tumor-related signature proteins in breast cancer captured by the dye. We selected the lysate of the tumor and paracancerous tissues from five breast cancer patients, co-incubated the targeted IR-780-alkyne with the tissue lysates. After IR-780-alkyne covalently bound to cysteine residues within the protein microreactor and the alkyne group served as a site for subsequent click chemistry. Through a copper-catalyzed azide-alkyne cycloaddition (click reaction), a biotin group was specifically conjugated to the alkyne-labeled proteins [35]. Streptavidin affinity was fixed on agarose resin as a solid-phase carrier. The binding between biotin and streptavidin affinity was very strong, effectively capturing biotin-labeled complexes. The targeted probe-protein complex labeled with biotin was mixed with the streptavidin carrier and incubated for a period of time to ensure full binding between biotin and streptavidin. After incubation, unbound impurities and non-specifically bound proteins were removed through washing steps. Finally, the tumor signature proteins were released from the streptavidin resin by elution, and dye-labeled tumor signature proteins were obtained (Fig. 2a and b).

We used liquid chromatography-mass spectrometry (LC-MS) to systematically characterize the tumor signature proteins of all NIR-FPs formed in our system. The results collectively suggested that IR-780-alkyne preferentially captured tumor-related signature proteins, highlighting its tumor-targeting selectivity in proteomic labeling (Fig. 2c and d). Mass spectrometric analysis revealed that among proteins with their molecular weights ranging from 15 to 100 kDa and exhibiting a fold change (FC) > 2 between tumor and paracancerous tissues, we identified a set of 20 tumor-related proteins (Fig. S11a). These proteins were reported in previous studies to promote cancer growth, migration, survival, or metastasis. Based on the 20 tumor-associated proteins identified by LC-MS in the preliminary stage, we further screened out 6 highly related proteins to tumors, including CCT5 (T-complex protein 1 subunit epsilon, encoded by *CCT5*) [[36], [37], [38], [39]], CPNE1 (Copine-1, encoded by *CPNE1*) [[40], [41], [42], [43], [44], [45]], FUS (Fused in sarcoma, encoded by *FUS*) [[46], [47], [48], [49], [50], [51]], HSPA4 (Heat shock 70 kDa protein 4, encoded by *HSPA4*) [[52], [53], [54], [55], [56]], ENO1 (Alpha-enolase, encoded by *ENO1*) [[57], [58], [59], [60], [61], [62]], and EIF5A (Eukaryotic translation initiation factor 5A-1, encoded by *EIF5A*) [[63], [64], [65], [66]], through GO annotation and evidence from previous studies linking them to tumor progression (Fig. 2e and Fig. S10). They may participate in the metabolic reprogramming, proliferation, and migration of cancer cells through a variety of mechanisms, and have the potential to serve as specific biomarkers for tumors. As shown in Fig. 2e, all of the proteins possess hydrophobic binding pockets containing accessible cysteine residues, which provide the reactive thiol groups and spatial environment necessary to undergo nucleophilic substitution reactions with IR-780-alkyne.

### 2.5 Verification of covalent binding between IR-780-alkyne and recombinant tumor signature proteins

**Fig. 3:**
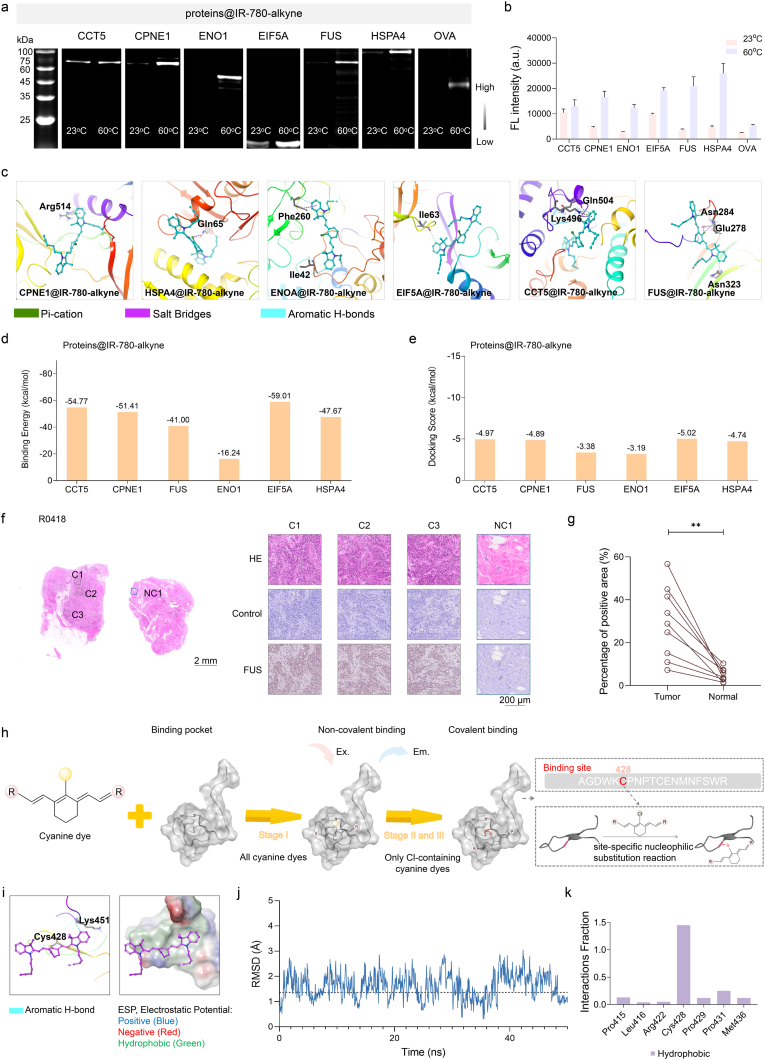
**Verification of covalent binding between IR-780-alkyne and recombinant tumor signature proteins.** (**a**) The tumor signature proteins were extracted and purified by the prokaryotic expression system, co-incubated with the dye and (**b**) the brightness statistics after co-incubation. (**c**) Theoretical simulation of IR-780-alkyne binding to CCT5, CPNE1, FUS, ENO1, EIF5A and HSPA4 proteins by docking mode. Comparison of (**d**) binding energy and (**e**) docking score between IR-780-alkyne and above six proteins. (**f**) Histological and immunohistochemical analysis of breast tumor tissue (R0418). H&E staining, negative control, and IHC staining for FUS were performed on adjacent sections of the same tumor region. (**g**) Quantitative comparison of FUS-positive areas in tumor and normal tissues (n = 9). (**h**) Mechanism of interaction between dyes and the FUS protein. First, all cyanine dyes insert into the hydrophobic binding pocket of FUS through noncovalent interactions. Then, only Cl-containing dyes (*e.g.*, IR-780-alkyne) underwent a site-specific nucleophilic substitution reaction with the thiol group of cysteine, forming a covalent bond. Stage III involves subsequent local conformational adjustment to yield a stable, highly emissive NIR-FPs. The 3D structural analysis revealed the binding site of Cl-containing dyes. The cysteine residue Cys428 in the binding pocket was confirmed as the covalent binding site responsible for Stage II. Ex: excitation; Em: emission. (**i**) Conformations of covalently docked FUS@IR-780-alkyne at steady state. (**j**) Dynamics simulation analysis of FUS@IR-780-alkyne covalent docking. (**k**) During the kinetic simulation, the contribution fraction of amino acid was evaluated together with their interaction with IR-780-alkyne.

To study the interaction between IR-780-alkyne and proteins, we extracted and purified the selected tumor signature proteins with a prokaryotic expression system. We first confirmed the labeling ability of IR-780-alkyne to recombinant tumor signature proteins (Fig. 3a). After co-incubation, these recombinant proteins had good covalent binding with IR-780-alkyne (Fig. 3b). When analyzing the covalent binding efficiency of the dye with proteins under 60^°^C incubation conditions, the brightness of the dye was significantly amplified several times, effectively confirming the overexpressed tumor signature proteins to accurately identify the tumor margin.

To further investigate the formation of NIR-FPs, we simulated the clear crystal structure of above-mentioned recombinant tumor signature proteins to analyze the binding principle between IR-780-alkyne and proteins (Fig. 3c and Fig. S12). Non-covalent interaction sites, involving key amino acid residues such as Asn284, Glu278 and Asn323, which interacted with IR-780-alkyne through hydrogen bonds, ion bonds, *etc*. Combined with its cysteine-mediated covalent binding site, the hydrophobic cavity of the FUS protein might have a matched binding space, thus giving it a high affinity. In contrast, the non-covalent interaction sites of ENO1 protein were relatively limited, including only Phe260 and Ile42, which might be related to its smaller hydrophobic cavity structure. To quantify the strength of these bindings, we used the Molecular Mechanics-Generalized Born Surface Area (MM-GBSA) method to calculate the docking score and binding energy of IR-780-alkyne and each protein (Fig. 3d and e). The results showed that the EIF5A protein and IR-780-alkyne had the highest docking score and the most binding energy, indicating that the bond between the two was the most stable. In contrast, the docking score and binding energy of ENO1 protein were the lowest, indicating that its binding with dye was weaker. It is worth noting that the binding energy only reflects the static intensity of the intermolecular interaction. In the actual tumor detection system, the binding contribution of protein also needs to be comprehensively judged in combination with its expression level in tumor tissue, the synergistic mode with dye, and the signal output ability. In general, in addition to the covalent binding, other supramolecular interactions between dye and proteins can enhance the brightness of the dye and promoted tumor detection.

Based on the target proteins (60-75 kDa) specifically labeled by IR-780-alkyne in breast tumors, we hypothesized that FUS (70 kDa) may contribute a substantial proportion of the ROI signals at 60 °C. Moreover, in AlphaFold3, the predicted interaction between this protein model and IR-780-alkyne exhibited the highest confidence score (Ranking score = 1.07) among all analyzed models (Fig. S11b). This protein has been previously reported to play critical roles in breast cancer initiation, progression, and therapeutic response, and it possesses the ability to form covalent bonds with IR-780-alkyne. We employed immunohistochemical (IHC) staining to assess FUS expression in tumor and normal tissues from nine breast cancer patients. Results showed that FUS expression was upregulated in tumor regions, confirming that this signal component could distinguish breast cancer tissues (Fig. 3f,g and Fig. S13). To elucidate the reaction mechanism between FUS and IR-780-alkyne, we performed proteomic analysis (Fig. 3h). NIR-FP (FUS@IR-780-alkyne) was first digested with chymotrypsin and trypsin, cleaving specific sites to peptides (Fig. S14). The resulting peptides were analyzed using HPLC-MS. Raw MS files were searched against a target protein database using BioPharma Finder 5.1 software, treating the tumor-selective dye as a variable modification (C42H48ClN2I, mass = 581.87 *m*/*z*). Detailed proteomic analysis confirmed that the dye-labeled peptide sequences corresponding to FUS residues Cys428 and Cys433 (Fig. S15). Notably, the Cys428 exhibited the highest confidence in identification analysis (Fig. S16). We predicted that the Cys428 residue is the primary covalent binding site between FUS and IR-780-alkyne. In addition, to further verify the mechanism of action, we performed covalent docking and molecular dynamics (MD) simulation (Fig. 3i–k) for the Cys428 site. The docking results revealed that IR-780-alkyne formed a stable bond at this site. The molecular dynamics trajectory showed that during the 50 ns simulation process, the RMSD of the complex remained within the stable range, suggesting that the FUS@IR-780-alkyne complex exhibited high stability in the overall configuration. Further interaction analysis demonstrated that hydrophobic interaction played a dominant role in the binding environment. Based on these computational results, we hypothesized that the cyanine dye interacted with the FUS protein through both noncovalent and covalent interactions, and the covalent complex between FUS and IR-780-alkyne underwent local conformational optimization to form a highly emissive and stable NIR-FP. It should be noted that the specific signal of ROI cannot be solely attributed to FUS. The identification of additional proteins is also the direction we will explore next, which will assist us in enhancing our understanding of the signature protein characteristics of breast tumors.

### 2.6 Tumor-to-paracancerous discrimination enabled by IR-780-alkyne

After establishing a reasonable tumor signature proteins-labeled mechanism, our next goal was to use clinical patient samples to evaluate their diagnostic ability. Due to the rapid proliferation of tumor cells, there was a large difference between the tumor microenvironment and normal tissue, which provided a potential advantage in distinguishing between tumor and paracancerous tissues. Based on the above experimental results, we further studied tumor/paracancerous tissues under different temperature and time conditions. The results showed that our dye could mark tumor signature proteins with different molecular weights in breast tumor tissue, while there were very few marked proteins in paracancerous tissue. The high fluorescence intensity ratio between tumor and paracancerous tissue (T/P ratio) was essential for tumor tissue identification. In addition, because temperature affected the binding efficacy between the dye and tumor signature protein, we also studied the fluorescence intensity of IR-780 and IR-780-alkyne after binding to tumor tissue lysate at different temperatures. The results showed that at various temperatures, the fluorescence intensity of IR-780-alkyne was higher than that of IR-780 (Fig. S17).

### 2.7 Enhanced microreactor: the improvement of temperature and reaction time on the tumor-positive edge discrimination effect

In our previous research, we expanded the traditional sodium dodecyl sulfate-polyacrylamide gel electrophoresis (SDS-PAGE) technology to the spatial mode to achieve a more in-depth analysis of proteins in tissue slices (Fig. S18) [[67], [68], [69]]. Using IR-780, we were able to quickly compare protein differences between benign and malignant tissues, thereby enhancing the ability to distinguish between tumors and normal tissues. During the research process, IR-780 demonstrated exceptional structural selective labeling ability of proteins, which laid the foundation for further research.

Based on these achievements, the current study focused on the development of an integrated NIR-FPs-based detection system, which combined spatially resolved protein analysis with the *in situ* formation of biomimetic NIR-FPs. In this system, tissue sections were labeled with tumor-selective dyes and subsequently separated electrophoretically according to protein molecular mass. To ensure effective separation within this system, the pore size of the gel was required to match the size screening mechanism of electrophoresis: proteins with larger molecular weights migrated more effectively in gels with larger pore sizes, while small molecules were better separated in gels with smaller pore sizes [70,71]. After separation, the gel layers containing the preselected proteins were fractionated and imaged to outline the tumor positive margins. To this end, we collected tumor specimens from breast cancer patients (R0274, R0573, and R0599) for subsequent experimental research. We analyzed tissue slices from patients labeled with IR-780 and IR-780 alkyne respectively, and quantified the fluorescence signals in tumor, paracancerous, and normal tissue areas. The experimental results showed that the performance of IR-780-alkyne was similar to that of IR-780, and in some cases, it was even more effective in distinguishing between tumors and paracancerous tissues (Fig. S19).

To further improve the evaluation accuracy of clinical tumor-positive edges, we took additional optimization steps to focus on the targeted staining conditions of “tumor signature protein@IR-780-alkyne FPs” in our system. Specifically, we collected tumor samples from five breast cancer patients, along with their corresponding paracancerous and normal tissues, and prepared these samples into frozen slices. Using the NIR-FPs-based detection system, we selected tissues from three patients for separation and analysis under different temperature and time conditions. Based on the molecular weight range (60-75 kDa) displayed by SDS-PAGE, we chose the corresponding z-direction positions (layer 3 to layer 5) for tumor detection analysis. The results from layers 3 to 10 reproduced the protein characteristics of tumor and paracancerous tissues (Fig. S20a,b, Fig. S21). After conducting three-dimensional histological electrophoresis and scanning the block layers, layers 3, 4, and 5 displayed the most clearly defined contours of tumor-positive regions. Therefore, we outlined the tumor contour in layers 3 or 4 using the NIR-FPs signals.

The quantitative analysis of the signal in the 8-layer cryosections (layer range: 3 to 10) of these five breast cancer samples showed a significant difference in the signal between tumor and paracancerous tissues. Further observations showed that in this system, the microreactor effect was enhanced as the temperature increased or the incubation time was moderately prolonged. However, in-depth analysis revealed that the tumor boundary recognition ability was optimal at 60^°^C and 60 min. In other words, although the microreactor effect continued to increase with temperature and time in the overall protein system, in the more complex tissue context, the optimal balance of detection sensitivity and specificity could only be achieved by accurately optimizing the reaction conditions. This optimization improved the effectiveness of the NIR-FPs-based detection system in distinguishing tumor positive margins (Fig. S20c).

Further experimental results showed that the NIR-FPs-based detection system could reliably evaluate tumor-positive edges with a high tumor-to-paracancer ratio under different temperature and time conditions (Fig. S20d and e). These results emphasized the effectiveness and sensitivity of the dye IR-780-alkyne, in clinical detection, and provided new tools for the rapid and accurate evaluation of tumor contours in the future.

### 2.8 Assessing the tumor-positive margins of patient samples using NIR-FPs

**Fig. 4:**
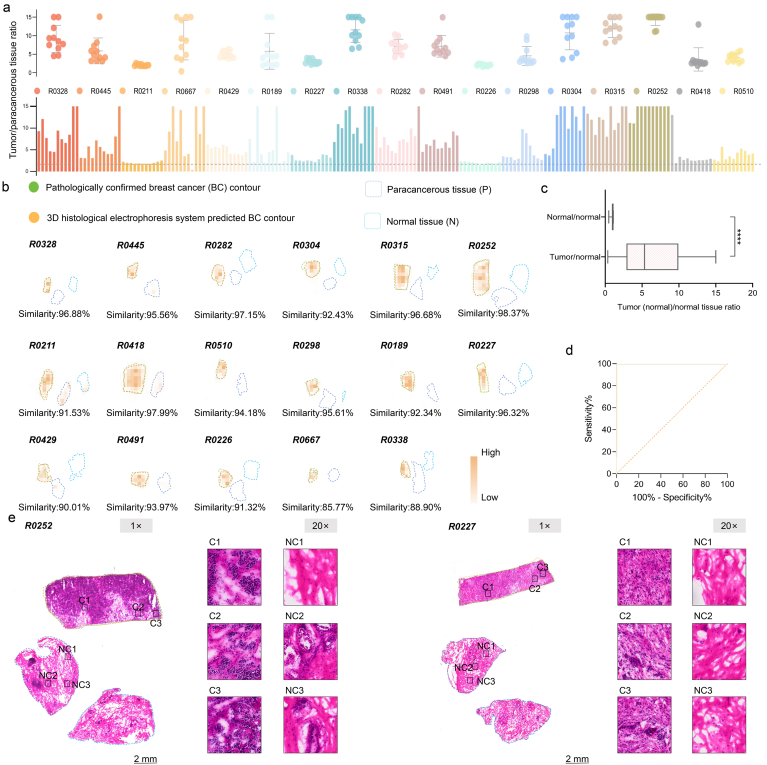
T**he NIR-FPs-based detection system was used to assess tumor-positive margins in breast cancer biopsies.** (**a**) Tumor-to-paracancerous tissue ratios (n = 11 for each biopsy). (**b**) The NIR-FPs-based detection system generated tumor contours, which were compared with pathologically confirmed contours, and the similarity between the two outcomes was calculated. (**c**) Comparison of the tumor-to-normal and normal-to-normal ratios from 11 fractionated layers. Significant differences were observed between tumor-positive and tumor-negative cohorts of breast cancer (∗∗∗∗*P* < 0.0001). (**d**) ROC curve analysis of the layers of tumor/normal and normal/normal sections was used to determine the cutoff (ratio = 1.330). (**e**) H&E staining was performed on breast cancer tumors (R0252 and R0227). Tumor-margin regions (C1-C3) identified by a pathologist were correctly included within the system-predicted tumor contours, while non-tumor regions (NC1-NC3) were excluded.

To prove that the NIR-FPs-based detection system could accurately evaluate clinical tumor-positive margins, patient breast cancer samples from 17 patients were collected together with the corresponding paracancerous and normal tissues. These tissues were made into frozen slices and then separated using our NIR-FPs-based detection system. Specifically, we divided the frozen slices of the 17 breast cancer samples into 11 fractions (from layer 3 to 13) and conducted signal quantitative analysis on these fractions. The results showed a significant signal difference between tumor tissue and paracancerous tissue. Our platform could robustly evaluate the tumor-positive margin with a high tumor-to-paracancer ratio (Fig. 4a) and compared the tumor-positive margins drawn by hematoxylin & eosin (H&E) (Fig. 4b). To further quantify the diagnostic performance of the NIR-FPs-based detection system in clinical samples, ROC curve analysis was performed using pathological diagnosis as the reference standard, and the corresponding results are presented in Fig. 4c and d.

To check the accuracy of our system, adjacent sections were simultaneously H&E-stained. An experienced pathologist was invited to examine these slices and delineate the cancer regions, with particular attention to representative boundary areas (*e.g.*, C1, C2, and C3). The three areas of our interest (C1, C2, and C3) were included in the system-predicted tumor contours, while the three non-tumor areas were located outside the predicted contours (Fig. 4e). This result demonstrated that the NIR-FPs-based detection system exhibited high sensitivity and accuracy in detecting and distinguishing tumor-positive edges.

In addition, the tumor contour predicted by the NIR-FPs-based detection system was highly consistent with the contour confirmed by pathologists, and the similarity of most quantitative comparisons was more than 90%. These results demonstrated that the NIR-FPs-based detection system could efficiently evaluate tumors and accurately predict their boundaries. It was especially noteworthy that this prediction was based on an objective analysis of quantitative signals, rather than relying on the subjective experience of pathologists. Compared with traditional clinical pathological examination, our NIR-FPs-based detection system could be operated by researchers without pathological experience, which greatly simplified the detection process and saved surgical time, thus supporting faster and more accurate intraoperative decision-making. This advantage made our system a potential alternative method, particularly in clinical situations that required real-time and reliable evaluation of tumor-positive margins.

### 2.9 The NIR-FPs-based detection system for detecting human breast cancer puncture samples

**Fig. 5:**
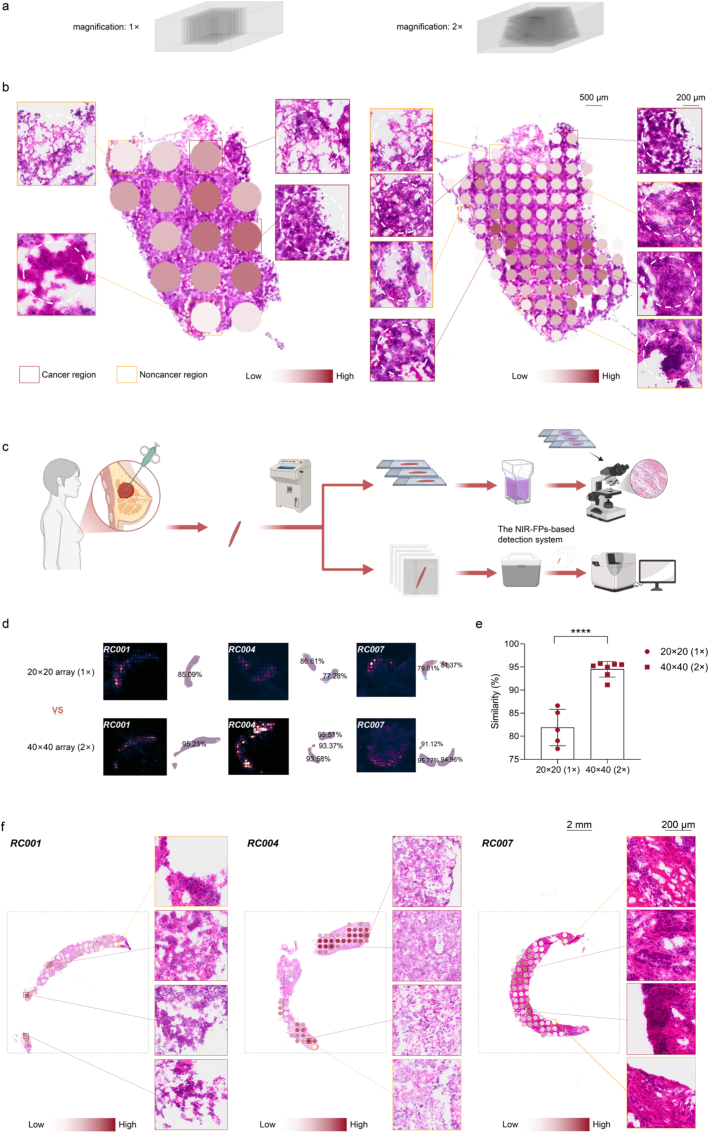
**The NIR-FPs-based detection system for detecting human breast cancer puncture samples.** (**a**) Microwell array molds for 3D histological electrophoresis. Close-up images of 20 × 20 and 40 × 40 microwell array molds. (**b**) Compared and analyzed the breast cancer tumor tissue using the 20 × 20 and the 40 × 40 microwell array module respectively. (**c**) Schematic diagram of the workflow of pathological examination and the NIR-FPs-based detection system of clinical puncture samples. (**d** and **e**) Representative fluorescence imaging-derived tumor profiles of clinical puncture samples predicted by the NIR-FPs-based detection system, together with the corresponding pathologically confirmed tumor profiles and the computational similarity between the two results under the 20 × 20 and 40 × 40 microwell arrays. (**f**) The breast puncture sample was stained with H&E, and the tumor area confirmed by pathology was compared with the tumor area predicted by the NIR-FPs-based detection system. Schemes were created with BioRender.com.

Through the above experiments, we verified that the tumor profile predicted by our NIR-FPs-based detection system was highly consistent with the contour confirmed by the pathologist. Among them, the similarity of most quantitative comparisons exceeded 90%, but there were still a small number of similarities below 90%, mainly concentrated in smaller tissue samples. The analysis showed that in the detection of smaller tissues, the microwell array in NIR-FPs-based detection system still had the issue of insufficient resolution. In response to this shortcoming, we improved the system and designed a module with amplification effect for the NIR-FPs-based detection system (Fig. 5a). We compared and analyzed the breast cancer tissue using the original 20 × 20 microwell array and the newly designed 40 × 40 microwell array respectively. The results showed that although both modules could accurately identify the contour of the tumor, the 40 × 40 microwell array had a higher resolution and could draw the contour of the tumor more finely (Fig. 5b).

Breast puncture was an important tool for the diagnosis of breast diseases, especially in the early detection of breast cancer [[72], [73], [74]]. To further improve the clinical applicability of the system, we applied the 40 × 40 microwell array to the detection of smaller tissues and selected clinical breast puncture samples for verification. We made these samples into frozen slices and separated them by the NIR-FPs-based detection system, while the adjacent slices were stained with H&E (Fig. 5c). Subsequently, an experienced pathologist was invited to examine the H&E staining section and outline the tumor area. In the end, we fitted and analyzed the tumor area outlined by the pathologist with the tumor profile predicted by our system. Experimental results showed that the diagnostic effect of the 40 × 40 microwell array in puncture samples was better than that of the 20 × 20 microwell array (Fig. 5d,e and Fig. S22). It was worth noting that the tumor area predicted by the system always included the cancer tissue area marked by the pathologist, and the non-tumor area was outside the predicted contour (Fig. 5f). This result showed that our NIR-FPs-based detection system had high sensitivity and accuracy in the detection and distinction of tumor-positive edges. Combined with the 40 × 40 microwell array, the NIR-FPs-based detection system could not only show good performance in the detection of routine surgical tissues but also have excellent diagnostic ability in the detection of smaller puncture tissues.

## 3 Conclusions

To achieve accurate targeting labeling, antibodies were typically conjugated to fluorescent molecules through linkers in traditional tumor immunohistochemistry. Although this method improved the accuracy of targeting to a certain extent, its development in clinical application was limited by many restrictions. First, the high cost of antibodies and the restricted scope of available antibody library have substantially limited the clinical intraoperative application of this strategy. Moreover, achieving accurate targeted imaging within complex biological systems depended on the immune interactions between multiple dye-labeled antibodies and tumor receptors (characteristic or signature proteins), as it was difficult for a single or a few active targeted receptors to show consistent effects in all cases. Compared with the traditional multicolor immunofluorescence staining method, the bionics-driven chemically selective protein labeling strategy we developed had the advantage of labeling a series of tumor signature proteins. It was not only simpler and faster, but also allowed labeling under mild physiological conditions without additional catalysts, thus providing more accurate test results.

We developed a novel chemogenic approach to create NIR-FPs for targeted tumor imaging by chemically labeling specific tumor signature proteins with dyes. The *in situ* formed “tumor signature protein@IR-780-alkyne FPs,” could highlight the tumor region with enhanced NIR brightness. This enhancement was due to the covalent binding facilitated by the protein's pocket conformation. We utilized IR-780-alkyne and proteomics to identify all tumor signature proteins that formed NIR-FPs in our system. By integrating three-dimensional histological electrophoresis with selected molecular weights of NIR-FPs, we effectively distinguished tumor tissues from paracancerous tissues in breast cancer samples. Optimizing the staining conditions further improved the system's accuracy in identifying tumor-positive margins, as confirmed through quantitative signal analysis and comparison with H&E staining. Our system demonstrated high sensitivity and accuracy in detecting tumor-positive edges, validated through a study of 17 clinical breast cancer samples. When used in conjunction with the high-throughput 40 × 40 microwell array module, this system demonstrated excellent diagnostic accuracy when processing small biopsy samples. This innovative approach has potential for enhancing the precision of cancer diagnostics and surgical margin assessments.

## CRediT authorship contribution statement

**Jianing Cheng:** Conceptualization, Data curation, Formal analysis, Writing – original draft. **Taiyu Liu:** Methodology. **Xintong Zhu:** Writing – review & editing. **Chengbin Zhang:** Methodology, Validation. **Yin Li:** Methodology. **Mengxin Li:** Resources. **Dong Song:** Funding acquisition, Resources, Supervision, Writing – review & editing. **Jia Li:** Conceptualization, Supervision, Writing – review & editing. **Feiran Zhang:** Conceptualization, Project administration, Supervision, Writing – review & editing. **Shoujun Zhu:** Conceptualization, Funding acquisition, Project administration, Supervision, Writing – review & editing.

## Declaration of competing interest

The authors declare that they have no known competing financial interests or personal relationships that could have appeared to influence the work reported in this paper.

## Data Availability

Data will be made available on request.
