# Supplementary material for: In situ biomimetic near-infrared fluorescent protein formed by tumor signature proteins for detecting breast cancer biopsy
Source: Mater Today Bio. 2026 Apr 27;38:103163. doi: 10.1016/j.mtbio.2026.103163 (PMC13153609; doi:10.1016/j.mtbio.2026.103163)
Supplement: Multimedia component 1 [file mmc1.docx]

Supplementary Information

***In situ* biomimetic near-infrared fluorescent protein formed by tumor signature proteins for detecting breast cancer biopsy**

Jianing Cheng^a,b^, Taiyu Liu^c^, Xintong Zhu^a^, Chengbin Zhang^d^, Yin Li^a,b^, Mengxin Li^e^, Dong Song^e^*, Jia Li^b^*, Feiran Zhang^b^*, Shoujun Zhu^a,b^*

^a^ State Key Laboratory of Supramolecular Structure and Materials, Center for Supramolecular Chemical Biology, College of Chemistry, Jilin University, Changchun 130012, P.R. China.

^b^ Joint Laboratory of Opto-Functional Theranostics in Medicine and Chemistry, The First Hospital of Jilin University, Changchun 130021, P.R. China.

^c^ The School of Pharmaceutical Sciences, Jilin University, 1266 Fujin Road, Changchun, Jilin 130021, P.R. China.

^d^ Department of Pathology, The First Hospital of Jilin University, Changchun 130021, P. R. China.

^e^ Department of Breast Surgery, General Surgery Center, The First Hospital of Jilin University, Changchun 130021, P. R. China.

***Corresponding author**

*Dong Song: Email: [songdong@jlu.edu.cn](mailto:songdong@jlu.edu.cn)

*Jia Li: Email: [lijia0204@jlu.edu.cn](mailto:lijia0204@jlu.edu.cn)

*Feiran Zhang: Email: [zhangfr@jlu.edu.cn](mailto:zhangfr@jlu.edu.cn)

*Shoujun Zhu: Email: [sjzhu@jlu.edu.cn](mailto:sjzhu@jlu.edu.cn)

**Contents**

[S1 Materials and Methods 3](#_Toc225874478)

[Materials. 3](#_Toc225874479)

[Clinical specimens and information. 3](#_Toc225874480)

[Ethical Statement. 4](#_Toc225874481)

[Lysis of tissues. 4](#_Toc225874482)

[Synthesis of IR-780-alkyne. 4](#_Toc225874483)

[Structural characterization and purity analysis of the dyes. 5](#_Toc225874484)

[Structural and purity characterization of IR-3B3. 6](#_Toc225874485)

[Structural and purity characterization of IR-780-alkyne. 6](#_Toc225874486)

[Structural and purity characterization of IR-780-alkyne-AC. 6](#_Toc225874487)

[Structural and purity characterization of IR-6B3C. 6](#_Toc225874488)

[Structural and purity characterization of IR-6B3S. 7](#_Toc225874489)

[Synthesis of NIR fluorescent proteins. 7](#_Toc225874490)

[The NIR-FPs-based detection system for comparative analysis of IR-780 and IR-780-alkyne labeled tissue slices. 7](#_Toc225874491)

[Microwell array mold manufacturing. 9](#_Toc225874492)

[H&E staining and imaging. 9](#_Toc225874493)

[Sodium dodecyl sulfate-polyacrylamide gel electrophoresis (SDS-PAGE) analysis and gel imaging. 9](#_Toc225874494)

[Immunohistochemical staining and imaging. 10](#_Toc225874495)

[Expression and purification of recombinant proteins. 10](#_Toc225874496)

[Preparation of recombinant proteins. 11](#_Toc225874497)

[Protein labeling and enrichment. 27](#_Toc225874498)

[Proteomics technique. 27](#_Toc225874499)

[Statistical analyses. 28](#_Toc225874500)

[S2 Supplementary Tables 29](#_Toc225874501)

[S3 Supplementary Figures 31](#_Toc225874502)

# S1 Materials and Methods

Materials. HSA, OVA, anhydrous dimethyl sulfoxide (DMSO, ≥ 99%), IR-780, IR-775, IR-783, ICG, ammonium bicarbonate, dithiothreitol (DTT), iodoacetamide (IAA), Tween-20, and tris[(1-benzyl-1H-1,2,3-triazol-4-yl)methyl]amine (TBTA) were purchased from Sigma-Aldrich. IR-808 was purchased from Adamas. Trypsin-EDTA was purchased from Gibco. Formic acid and acetonitrile were purchased from Thermo Fisher. Ni-NTA agarose (His-Tag), 50×TAE buffer (Tris-acetate-EDTA), agar, imidazole, LB broth powder, phenylmethylsulfonyl fluoride (PMSF), kanamycin sulfate, ampicillin sodium, lysozyme, and IPTG were purchased from Sangon Biotech. Sodium dodecyl sulfate (SDS), glycine, Tris base, tris(2-carboxyethyl)phosphine hydrochloride (TCEP·HCl), and sodium chloride (NaCl) were purchased from Yeasen. Biotin-PEG4-azide was purchased from TCI. BeyoZonase Super Nuclease was purchased from Beyotime.

Clinical specimens and information. All human breast cancer specimens analyzed in this study were obtained from the Department of Biobank, Division of Clinical Research, The First Hospital of Jilin University. Taking the collection process of breast cancer cases as an example, postoperative specimens of breast cancer patients were stored at -80^o^C until analysis. The surgical specimens were embedded in the optimal cutting temperature (OCT, SAKYRA) compound, cryosectioned into 60 µm-thick slices (Leica, 39475237), and immediately subjected to histological electrophoresis analysis using the NIR-FPs-based detection system. Clinical information, including TNM staging and histological subtype, was collected and analyzed from the pathological reports of these patients.

Ethical Statement. Human specimens were obtained from the Department of Biobank, Division of Clinical Research, the First Hospital of Jilin University. All experiments involving human specimens were approved by the Ethics Committee of the First Hospital of Jilin University (2023-284-1).

Lysis of tissues. Cut the tissue into sufficiently small pieces and add an appropriate amount of RIPA buffer (Biosharp) into the Eppendorf tube containing the tissue fragments. To ensure sufficient lysis, the tube was sonicated for 15 min at 4^o^C under a contact-free sonicator with 80% power density. The lysed tissue was centrifuged for 10 min at 13000 g, allowing the remaining fragments to pellet at the bottom of the tube. Finally, the supernatant (lysis solution) was collected and protein concentration was measured using a NanoDrop spectrophotometer.

Synthesis of IR-780-alkyne. Compound 1 was synthesized by adding 2,3,3-trimethyl-3H-indole (1.50 mL, 9.34 mmol) and anhydrous acetonitrile (10 mL) into a sealed reaction vessel, followed by the addition of 6-iodo-1-hexyne (1.00 mL, 7.70 mmol). The reaction mixture was heated and stirred until completion. After cooling to room temperature, ethyl acetate was added to precipitate the product. The resulting light gray solid was collected by filtration to afford compound 1 in a several-hundred-milligram scale with a yield of approximately 40%. IR-780-alkyne was then synthesized by adding compound 1 (400 mg, 1.10 mmol), 2-chloro-3-(hydroxymethylene)-1-cyclohexene-1-carbaldehyde (100 mg, 0.54 mmol), and sodium acetate (160 mg, 1.80 mmol) into a reaction flask, followed by the addition of acetic anhydride (20 mL). The reaction mixture was stirred at approximately 35^o^C until completion (about 3-5 h). After the reaction was completed, the solvent was removed under reduced pressure. The resulting residue was purified by silica gel column chromatography using dichloromethane/methanol as the eluent (for example, 100:1, v/v) to afford IR-780-alkyne as a green solid in approximately 70% yield.


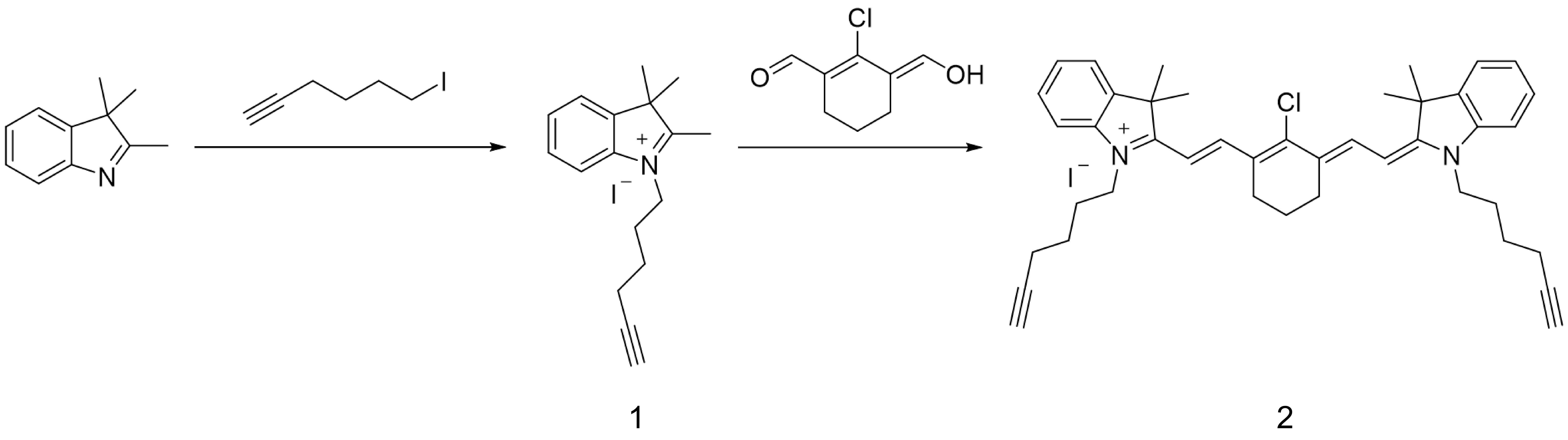


Scheme S1. Synthetic route of IR-780-alkyne.

Structural characterization and purity analysis of the dyes. ^1^H-NMR spectra were recorded on a Bruker AVANCE III 400 MHz NMR spectrometer (Q. One Instruments Ltd.). Chemical shift multiplicities are denoted as s (singlet), d (doublet), t (triplet), and m (multiplet), and coupling constants (J) are reported in Hz. Mass spectrometric analyses were carried out using a QSTAR Elite instrument (ABI). High-resolution LC/MS measurements were performed under the following conditions: electrospray ionization in positive mode at 4.5 kV or in negative mode at -3.5 kV; nebulizing gas flow, 1.5 L/min; drying gas pressure, 100 kPa; heat block temperature, 200^o^C; CDL temperature, 200^o^C; ion trap vacuum, 1.0×10^-2^ Pa; and TOF vacuum, 5×10^-4^ Pa.

Structural and purity characterization of IR-3B3. ^1^H-NMR (400 MHz, DMSO-*d_6_*) *δ* 8.28 (d, *J* = 14.1 Hz, 2H), 7.64 (dd, *J* = 7.5, 1.2 Hz, 2H), 7.52 – 7.38 (m, 4H), 7.29 (td, *J* = 7.3, 1.2 Hz, 2H), 6.35 (d, *J* = 14.2 Hz, 2H), 4.20 (t, *J* = 7.2 Hz, 4H), 2.73 (t, *J* = 6.4 Hz, 4H), 1.89 – 1.82 (m, 2H), 1.78 (q, *J* = 7.2 Hz, 4H), 1.69 (s, 12H), 0.96 (t, *J* = 7.2 Hz, 6H).

Structural and purity characterization of IR-780-alkyne. ^1^H-NMR (400 MHz, CDCl_3_) *δ* 8.34 (d, *J* = 14.0 Hz, 2H), 7.42 – 7.37 (m, 4H), 7.23 – 7.20 (m, 4H), 6.30 (d, *J* = 14.4 Hz, 2H), 4.29 (t, *J* = 7.6 Hz, 4H), 2.78 (t, *J* = 6.0 Hz, 4H), 2.36 – 2.32 (m, 4H), 2.05 – 1.97 (m, 8H), 1.79 – 1.73 (m, 16H).

Structural and purity characterization of IR-780-alkyne-AC. ^1^H-NMR (400 MHz, CD_3_OD) *δ* 8.81 (d, *J* = 14.1 Hz, 2H), 7.51 (dd, *J* = 7.6, 1.2 Hz, 2H), 7.41 (td, *J* = 7.6, 1.2 Hz, 2H), 7.31 (d, *J* = 8.0 Hz, 2H), 7.26 (t, *J* = 7.2 Hz, 2H), 6.29 (d, *J* = 14.0 Hz, 2H), 4.42 – 4.33 (m, 1H), 4.18 (t, *J* = 7.6 Hz, 4H), 3.49 – 3.40 (m, 1H), 3.27 – 3.18 (m, 1H), 2.80 – 2.58 (m, 4H), 2.43 – 2.22 (m, 6H), 2.01 – 1.91 (m, 9H), 1.76 (s, 12H), 1.72 – 1.65 (m, 4H).

Structural and purity characterization of IR-6B3C. ^1^H-NMR (400 MHz, DMSO-*d_6_*) *δ* 8.24 (d, *J* = 14.0 Hz, 2H), 7.62 (d, *J* = 7.2 Hz, 2H), 7.49 – 7.38 (m, 4H), 7.28 (t, *J* = 8.0 Hz, 2H), 6.42 (d, *J* = 14.0 Hz, 2H), 4.43 (t, *J* = 7.2 Hz, 4H), 2.78 – 2.64 (m, 8H), 1.92 – 1.79 (m, 2H), 1.66 (s, 12H).

Structural and purity characterization of IR-6B3S. ^1^H NMR (400 MHz, DMSO-*d_6_*) *δ* 8.26 (d, *J* = 14.0 Hz, 1H), 7.62 (dd, *J* = 7.5, 1.2 Hz, 2H), 7.54 (d, *J* = 8.0 Hz, 2H), 7.42 (td, *J* = 7.6, 1.2 Hz, 2H), 7.27 (td, *J* = 7.4, 0.9 Hz, 2H), 6.53 (d, *J* = 14.1 Hz, 2H), 4.38 (t, *J* = 7.6 Hz, 4H), 2.75 (t, *J* = 6.1 Hz, 4H), 2.57 (t, *J* = 6.7 Hz, 4H), 2.03 (p, *J* = 7.2 Hz, 4H), 1.89 – 1.78 (d, *J* = 6.6 Hz, 2H), 1.68 (s, 12H).

Synthesis of NIR fluorescent proteins. All NIR fluorescent proteins were synthesized in a similar way. First, the breast cancer tissue was lysed to obtain the human breast cancer tissue lysate, which was diluted to a concentration of 10 µmol. Then, 2 mmol IR-780-alkyne was prepared in anhydrous dimethyl sulfoxide (DMSO). Next, IR-780-alkyne (final concentration 2 mmol) was added to the 10 μmol of breast cancer tissue lysate (maintaining a protein-to-dye molar ratio of 1:2), and the mixture was thoroughly mixed by rapid vortexing. The mixture was then incubated at 60^o^C with shaking for 2 hours to obtain the NIR-FPs.

The NIR-FPs-based detection system for comparative analysis of IR-780 and IR-780-alkyne labeled tissue slices. Proteins in frozen slices were labeled and separated by the methods described above. The Frozen tissue slices (thickness, 60 µm) were adhered to the microwell array molds (optical cross-linking resin), and then subjected to lysis and labeling. To dissociate and label simultaneously, tissue slices were incubated in a culture dish (diameter, 5 cm) containing a dye (IR-780 and IR-780-alkyne) solution [5 mL, 1 µmol radioimmunoprecipitation assay (RIPA) buffer] for 30 minutes, with the tissue side facing downward to ensure direct contact with the lysis and labeling solution. After lysis and labeling, the non-crosslinked stacking gel was poured onto the top of the cross-linked separation gel in the electrophoresis tank. The array mold (with the tissue slice facing upward) was then immediately and carefully placed into the non-crosslinked stacking gel. The stacking gel entered the micropore from the side without tissue slices and cross-linked to form a stacked gel column. To better isolate the proteins of interest in tissue according to molecular mass, a 10% polyacrylamide gel (PAG) was selected for three-dimensional tissue electrophoresis of tissue slices. After the stacking gel was cross-linked within the micropores to form stacked gel columns, 1× electrophoresis buffer was carefully added, and the proteins were separated by electrophoresis at 40 V for 10 minutes. After three-dimensional tissue electrophoresis, the proteins in the tissue were separated, and their histological information was preserved. The electrophoretic gel containing protein was completely frozen at -80^o^C and then separated into layers with a thickness of 600 µm. The separated gel layers containing the target protein signal were then imaged with a NIR scanner (Azure, Sapphire) at the 800 nm channel, with a specific fluorescence intensity and a pixel size of 100 µm.

Microwell array mold manufacturing. The microwell array molds were designed using 3D modeling software (AutoCAD 2020) and designed with two different throughputs (20×20 and 40×40). To provide adequate electrophoretic distance for effective protein concentration, the thickness of all molds was set to 0.5 cm. The molds were fabricated with a 3D printer (Anycubic, Photon Mono X) using photocrosslinking resin (UV resin, 1 kg; Anycubic) under an exposure time of 2 s. After printing, the molds were subjected to sonication twice in 99% ethanol, each for 3 min.

H&E staining and imaging. All tissue slices (thickness: 15 µm) were stored at -20^o^C until staining. Automated H&E staining was performed using a Leica SPIRIT ST-CV5030 automatic stainer. Slides underwent fixation, hematoxylin staining, differentiation, bluing, and eosin counterstaining steps according to optimized, preset conditions. After staining, slides were automatically dehydrated, cleared, and coverslipped. Olympus full-slide scanning system (VS200) was used to obtain H&E stained images of the tissue.

Sodium dodecyl sulfate-polyacrylamide gel electrophoresis (SDS-PAGE) analysis and gel imaging. SDS-PAGE analysis was performed with a commercial kit (Yeasen). Tissue lysates labeled with dye were combined with 5× protein loading buffer. Following sample preparation, electrophoresis buffer was added, gels were run at 80 V for stacking and 120 V for separation on a BIO-RAD electrophoresis system (USA). The run was stopped when bromophenol blue migrated to within 1 cm of the gel bottom. Subsequently, the gels were scanned at the 800 nm channel using a fluorescence scanner (Sapphire, Azure) with defined intensity and a pixel resolution of 100 µm. Fluorescence signals were quantified from the acquired images using ImageJ software.

Immunohistochemical staining and imaging. Paraffin-embedded human breast cancer tissues were sectioned at 3.5 μm and baked at 65 °C for 1 h. Slides were deparaffinized with xylene and rehydrated through graded ethanol solutions. Antigen retrieval was performed using Tris-EDTA buffer (pH~9.0) in a microwave oven. Endogenous peroxidase activity was blocked, followed by incubation with 5% BSA for 30 min at room temperature. The primary antibody was rabbit monoclonal anti-TLS/FUS (ab124923, Abcam), diluted 1:800 and incubated overnight at 4^o^C. After washing, horseradish peroxidase (HRP) conjugated goat anti-rabbit secondary antibodies were applied. Signal detection was achieved using DAB substrate (~20 s). Nuclei were counterstained with hematoxylin, and slides were dehydrated, cleared, and coverslipped for imaging.

Expression and purification of recombinant proteins. Candidate genes were cloned into either the pET-28a (+) or pCold-II expression vector and transformed into *E. coli* Rosetta (DE3) competent cells. Transformed colonies were cultured overnight in LB medium supplemented with the appropriate antibiotic (kanamycin for pET-28a(+), ampicillin for pCold-II). The overnight culture was diluted 1:80 into fresh LB medium and grown at 37^o^C until OD_600_ reached 0.6-0.8. For pET-28a (+) constructs, protein expression was induced with 0.4 mmol IPTG and carried out at 25-30^o^C for 6-12 h. For pCold-II constructs, induction was performed with 0.2 mmol IPTG at 15^o^C for 24 h. Bacterial pellets were harvested by centrifugation and stored at -20^o^C or -80^o^C for further processing. Bacterial pellets were resuspended in lysis buffer and treated with lysozyme, nuclease, protease inhibitors, and detergent. After incubation to reduce viscosity, lysates were centrifuged, and the supernatant was collected as the soluble protein fraction. His-tagged recombinant proteins were purified by nickel-affinity chromatography using Ni^2+^-charged agarose resin. Following incubation with the lysate, the resin was washed with buffers containing increasing concentrations of imidazole to remove nonspecific proteins. Target proteins were eluted with high-imidazole elution buffer in stepwise gradients. The purity of the eluted proteins was assessed by SDS-PAGE followed by Coomassie Brilliant Blue staining (20309ES03, Yeasen).

Preparation of recombinant proteins. To investigate the biological functions of candidate proteins, several recombinant human proteins, including CCT5, CPNE1, FUS, HSPA4, ENO1, and EIF5A, were generated. All constructs were verified by DNA sequencing prior to expression. The nucleotide and corresponding amino acid sequences used for recombinant protein expression are listed below.

1. The DNA sequence of CCT5:

ATGGCGTCCATGGGGACCCTCGCCTTCGATGAATATGGGCGCCCTTTCCTCATCATCAAGGATCAGGACCGCAAGTCCCGTCTTATGGGACTTGAGGCCCTCAAGTCTCATATAATGGCAGCAAAGGCTGTAGCAAATACAATGAGAACATCACTTGGACCAAATGGGCTTGATAAGATGATGGTGGATAAGGATGGGGATGTGACTGTAACTAATGATGGGGCCACCATCTTAAGCATGATGGATGTTGATCATCAGATTGCCAAGCTGATGGTGGAACTGTCCAAGTCTCAGGATGATGAAATTGGAGATGGAACCACAGGAGTGGTTGTCCTGGCTGGTGCCTTGTTAGAAGAAGCGGAGCAATTGCTAGACCGAGGCATTCACCCAATCAGAATAGCCGATGGCTATGAGCAGGCTGCTCGCGTTGCTATTGAACACCTGGACAAGATCAGCGATAGCGTCCTTGTTGACATAAAGGACACCGAACCCCTGATTCAGACAGCAAAAACCACGCTGGGCTCCAAAGTGGTCAACAGTTGTCACCGACAGATGGCTGAGATTGCTGTGAATGCCGTCCTCACTGTAGCAGATATGGAGCGGAGAGACGTTGACTTTGAGCTTATCAAAGTAGAAGGCAAAGTGGGCGGCAGGCTGGAGGACACTAAACTGATTAAGGGCGTGATTGTGGACAAGGATTTCAGTCACCCACAGATGCCAAAAAAAGTGGAAGATGCGAAGATTGCAATTCTCACATGTCCATTTGAACCACCCAAACCAAAAACAAAGCATAAGCTGGATGTGACCTCTGTCGAAGATTATAAAGCCCTTCAGAAATACGAAAAGGAGAAATTTGAAGAGATGATTCAACAAATTAAAGAGACTGGTGCTAACCTAGCAATTTGTCAGTGGGGCTTTGATGATGAAGCAAATCACTTACTTCTTCAGAACAACTTGCCTGCGGTTCGCTGGGTAGGAGGACCTGAAATTGAGCTGATTGCCATCGCAACAGGAGGGCGGATCGTCCCCAGGTTCTCAGAGCTCACAGCCGAGAAGCTGGGCTTTGCTGGTCTTGTACAGGAGATCTCATTTGGGACAACTAAGGATAAAATGCTGGTCATCGAGCAGTGTAAGAACTCCAGAGCTGTAACCATTTTTATTAGAGGAGGAAATAAGATGATCATTGAGGAGGCGAAACGATCCCTTCACGATGCTTTGTGTGTCATCCGGAACCTCATCCGCGATAATCGTGTGGTGTATGGAGGAGGGGCTGCTGAGATATCCTGTGCCCTGGCAGTTAGCCAAGAGGCGGATAAGTGCCCCACCTTAGAACAGTATGCCATGAGAGCGTTTGCCGACGCACTGGAGGTCATCCCCATGGCCCTCTCTGAAAACAGTGGCATGAATCCCATCCAGACTATGACCGAAGTCCGAGCCAGACAGGTGAAGGAGATGAACCCTGCTCTTGGCATCGACTGTTTGCACAAGGGGACAAATGATATGAAGCAACAGCATGTCATAGAAACCTTGATTGGCAAAAAGCAACAGATATCTCTTGCAACACAAATGGTTAGAATGATTTTGAAGATTGATGACATTCGTAAGCCTGGAGAATCTGAAGAA.

The amino acid sequence of CCT5: (541 AAs; 59.67 kDa)

MASMGTLAFDEYGRPFLIIKDQDRKSRLMGLEALKSHIMAAKAVANTMRTSLGPNGLDKMMVDKDGDVTVTNDGATILSMMDVDHQIAKLMVELSKSQDDEIGDGTTGVVVLAGALLEEAEQLLDRGIHPIRIADGYEQAARVAIEHLDKISDSVLVDIKDTEPLIQTAKTTLGSKVVNSCHRQMAEIAVNAVLTVADMERRDVDFELIKVEGKVGGRLEDTKLIKGVIVDKDFSHPQMPKKVEDAKIAILTCPFEPPKPKTKHKLDVTSVEDYKALQKYEKEKFEEMIQQIKETGANLAICQWGFDDEANHLLLQNNLPAVRWVGGPEIELIAIATGGRIVPRFSELTAEKLGFAGLVQEISFGTTKDKMLVIEQCKNSRAVTIFIRGGNKMIIEEAKRSLHDALCVIRNLIRDNRVVYGGGAAEISCALAVSQEADKCPTLEQYAMRAFADALEVIPMALSENSGMNPIQTMTEVRARQVKEMNPALGIDCLHKGTNDMKQQHVIETLIGKKQQISLATQMVRMILKIDDIRKPGESEE.

The amino acid sequence fused with His tag (His tag-CCT5):

HHHHHHMASMGTLAFDEYGRPFLIIKDQDRKSRLMGLEALKSHIMAAKAVANTMRTSLGPNGLDKMMVDKDGDVTVTNDGATILSMMDVDHQIAKLMVELSKSQDDEIGDGTTGVVVLAGALLEEAEQLLDRGIHPIRIADGYEQAARVAIEHLDKISDSVLVDIKDTEPLIQTAKTTLGSKVVNSCHRQMAEIAVNAVLTVADMERRDVDFELIKVEGKVGGRLEDTKLIKGVIVDKDFSHPQMPKKVEDAKIAILTCPFEPPKPKTKHKLDVTSVEDYKALQKYEKEKFEEMIQQIKETGANLAICQWGFDDEANHLLLQNNLPAVRWVGGPEIELIAIATGGRIVPRFSELTAEKLGFAGLVQEISFGTTKDKMLVIEQCKNSRAVTIFIRGGNKMIIEEAKRSLHDALCVIRNLIRDNRVVYGGGAAEISCALAVSQEADKCPTLEQYAMRAFADALEVIPMALSENSGMNPIQTMTEVRARQVKEMNPALGIDCLHKGTNDMKQQHVIETLIGKKQQISLATQMVRMILKIDDIRKPGESEE.

2. The DNA sequence of CPNE1:

atggcccactgcgtgaccttggttcagctgtccatttcctgtgaccatctcattgacaaggacatcggctccaagtctgacccactctgcgtccttttacaggatgtgggagggggcagctgggctgagcttggccggactgaacgggtgcggaactgctcaagccctgagttctccaagactctacagcttgagtaccgctttgagacagtccagaagctacgctttggaatctatgacatagacaacaagacgccagagctgagggatgatgacttcctagggggtgctgagtgttccctaggacagattgtgtccagccaggtactgactctccccttgatgctgaagcctggaaaacctgctgggcgggggaccatcacggtctcagctcaggaattaaaggacaatcgtgtagtaaccatggaggtagaggccagaaacctagataagaaggacttcctgggaaaatcagatccatttctggagttcttccgccagggtgatgggaaatggcacctggtgtacagatctgaggtcatcaagaacaacctgaaccctacatggaagcgtttctcagtccccgttcagcatttctgtggtgggaaccccagcacacccatccaggtgcaatgctccgattatgacagtgacgggtcacatgatctcatcggtaccttccacaccagcttggcccagctgcaggcagtcccggctgagtttgaatgcatccaccctgagaagcagcagaaaaagaaaagctacaagaactctggaactatccgtgtcaagatttgtcgggtagaaacagagtactcctttctggactatgtgatgggaggctgtcagatcaacttcactgtgggcgtggacttcactggctccaatggagacccctcctcacctgactccctacactacctgagtccaacaggggtcaatgagtacctgatggcactgtggagtgtgggcagcgtggttcaggactatgactcagacaagctgttccctgcatttggatttggggcccaggttccccctgactggcaggtctcgcatgaatttgccttgaatttcaaccccagtaacccctactgtgcaggcatccagggcattgtggatgcctaccgccaagccctgccccaagttcgcctctatggccctaccaactttgcacccatcatcaaccatgtggccaggtttgcagcccaggctgcacatcaggggactgcctcgcaatacttcatgctgttgctgctgactgatggtgctgtgacggatgtggaagccacacgtgaggctgtggtgcgtgcctcgaacctgcccatgtcagtgatcattgtgggtgtgggtggtgctgactttgaggccatggagcagctggacgctgatggtggacccctgcatacacgttctgggcaggctgctgcccgcgacattgtgcagtttgtaccctaccgccggttccagaatgcccctcgggaggcattggcacagaccgtgctcgcagaagtgcccacacaactggtctcatacttcagggcccagggttgggccccgctcaagccacttccaccctcagccaaggatcctgcacaggccccccaggcc.

The amino acid sequence of CPNE1: (537 AAs; 59.06 kDa)

MAHCVTLVQLSISCDHLIDKDIGSKSDPLCVLLQDVGGGSWAELGRTERVRNCSSPEFSKTLQLEYRFETVQKLRFGIYDIDNKTPELRDDDFLGGAECSLGQIVSSQVLTLPLMLKPGKPAGRGTITVSAQELKDNRVVTMEVEARNLDKKDFLGKSDPFLEFFRQGDGKWHLVYRSEVIKNNLNPTWKRFSVPVQHFCGGNPSTPIQVQCSDYDSDGSHDLIGTFHTSLAQLQAVPAEFECIHPEKQQKKKSYKNSGTIRVKICRVETEYSFLDYVMGGCQINFTVGVDFTGSNGDPSSPDSLHYLSPTGVNEYLMALWSVGSVVQDYDSDKLFPAFGFGAQVPPDWQVSHEFALNFNPSNPYCAGIQGIVDAYRQALPQVRLYGPTNFAPIINHVARFAAQAAHQGTASQYFMLLLLTDGAVTDVEATREAVVRASNLPMSVIIVGVGGADFEAMEQLDADGGPLHTRSGQAAARDIVQFVPYRRFQNAPREALAQTVLAEVPTQLVSYFRAQGWAPLKPLPPSAKDPAQAPQA.

The amino acid sequence fused with His tag (His tag-CPNE1):

HHHHHHMAHCVTLVQLSISCDHLIDKDIGSKSDPLCVLLQDVGGGSWAELGRTERVRNCSSPEFSKTLQLEYRFETVQKLRFGIYDIDNKTPELRDDDFLGGAECSLGQIVSSQVLTLPLMLKPGKPAGRGTITVSAQELKDNRVVTMEVEARNLDKKDFLGKSDPFLEFFRQGDGKWHLVYRSEVIKNNLNPTWKRFSVPVQHFCGGNPSTPIQVQCSDYDSDGSHDLIGTFHTSLAQLQAVPAEFECIHPEKQQKKKSYKNSGTIRVKICRVETEYSFLDYVMGGCQINFTVGVDFTGSNGDPSSPDSLHYLSPTGVNEYLMALWSVGSVVQDYDSDKLFPAFGFGAQVPPDWQVSHEFALNFNPSNPYCAGIQGIVDAYRQALPQVRLYGPTNFAPIINHVARFAAQAAHQGTASQYFMLLLLTDGAVTDVEATREAVVRASNLPMSVIIVGVGGADFEAMEQLDADGGPLHTRSGQAAARDIVQFVPYRRFQNAPREALAQTVLAEVPTQLVSYFRAQGWAPLKPLPPSAKDPAQAPQA.

3. The DNA sequence of FUS:

atggcctcaaacgattatacccaacaagcaacccaaagctatggggcctaccccacccagcccgggcagggctattcccagcagagcagtcagccctacggacagcagagttacagtggttatagccagtccacggacacttcaggctatggccagagcagctattcttcttatggccagagccagaacacaggctatggaactcagtcaactccccagggatatggctcgactggcggctatggcagtagccagagctcccaatcgtcttacgggcagcagtcctcctaccctggctatggccagcagccagctcccagcagcacctcgggaagttacggtagcagttctcagagcagcagctatgggcagccccagagtgggagctacagccagcagcctagctatggtggacagcagcaaagctatggacagcagcaaagctataatccccctcagggctatggacagcagaaccagtacaacagcagcagtggtggtggaggtggaggtggaggtggaggtaactatggccaagatcaatcctccatgagtagtggtggtggcagtggtggcggttatggcaatcaagaccagagtggtggaggtggcagcggtggctatggacagcaggaccgtggaggccgcggcaggggtggcagtggtggcggcggcggcggcggcggtggtggttacaaccgcagcagtggtggctatgaacccagaggtcgtggaggtggccgtggaggcagaggtggcatgggcggaagtgaccgtggtggcttcaataaatttggtggccctcgggaccaaggatcacgtcatgactccgaacaggataattcagacaacaacaccatctttgtgcaaggcctgggtgagaatgttacaattgagtctgtggctgattacttcaagcagattggtattattaagacaaacaagaaaacgggacagcccatgattaatttgtacacagacagggaaactggcaagctgaagggagaggcaacggtctcttttgatgacccaccttcagctaaagcagctattgactggtttgatggtaaagaattctccggaaatcctatcaaggtctcatttgctactcgccgggcagactttaatcggggtggtggcaatggtcgtggaggccgagggcgaggaggacccatgggccgtggaggctatggaggtggtggcagtggtggtggtggccgaggaggatttcccagtggaggtggtggcggtggaggacagcagcgagctggtgactggaagtgtcctaatcccacctgtgagaatatgaacttctcttggaggaatgaatgcaaccagtgtaaggcccctaaaccagatggcccaggagggggaccaggtggctctcacatggggggtaactacggggatgatcgtcgtggtggcagaggaggctatgatcgaggcggctaccggggccgcggcggggaccgtggaggcttccgagggggccggggtggtggggacagaggtggctttggccctggcaagatggattccaggggtgagcacagacaggatcgcagggagaggccgtat.

The amino acid sequence of FUS: (526 AAs; 70 kDa)

MASNDYTQQATQSYGAYPTQPGQGYSQQSSQPYGQQSYSGYSQSTDTSGYGQSSYSSYGQSQNTGYGTQSTPQGYGSTGGYGSSQSSQSSYGQQSSYPGYGQQPAPSSTSGSYGSSSQSSSYGQPQSGSYSQQPSYGGQQQSYGQQQSYNPPQGYGQQNQYNSSSGGGGGGGGGGNYGQDQSSMSSGGGSGGGYGNQDQSGGGGSGGYGQQDRGGRGRGGSGGGGGGGGGGYNRSSGGYEPRGRGGGRGGRGGMGGSDRGGFNKFGGPRDQGSRHDSEQDNSDNNTIFVQGLGENVTIESVADYFKQIGIIKTNKKTGQPMINLYTDRETGKLKGEATVSFDDPPSAKAAIDWFDGKEFSGNPIKVSFATRRADFNRGGGNGRGGRGRGGPMGRGGYGGGGSGGGGRGGFPSGGGGGGGQQRAGDWKCPNPTCENMNFSWRNECNQCKAPKPDGPGGGPGGSHMGGNYGDDRRGGRGGYDRGGYRGRGGDRGGFRGGRGGGDRGGFGPGKMDSRGEHRQDRRERPY.

The amino acid sequence fused with His tag and Myc tag (His tag-FUS-Myc tag):

HHHHHHHHHHMASNDYTQQATQSYGAYPTQPGQGYSQQSSQPYGQQSYSGYSQSTDTSGYGQSSYSSYGQSQNTGYGTQSTPQGYGSTGGYGSSQSSQSSYGQQSSYPGYGQQPAPSSTSGSYGSSSQSSSYGQPQSGSYSQQPSYGGQQQSYGQQQSYNPPQGYGQQNQYNSSSGGGGGGGGGGNYGQDQSSMSSGGGSGGGYGNQDQSGGGGSGGYGQQDRGGRGRGGSGGGGGGGGGGYNRSSGGYEPRGRGGGRGGRGGMGGSDRGGFNKFGGPRDQGSRHDSEQDNSDNNTIFVQGLGENVTIESVADYFKQIGIIKTNKKTGQPMINLYTDRETGKLKGEATVSFDDPPSAKAAIDWFDGKEFSGNPIKVSFATRRADFNRGGGNGRGGRGRGGPMGRGGYGGGGSGGGGRGGFPSGGGGGGGQQRAGDWKCPNPTCENMNFSWRNECNQCKAPKPDGPGGGPGGSHMGGNYGDDRRGGRGGYDRGGYRGRGGDRGGFRGGRGGGDRGGFGPGKMDSRGEHRQDRRERPYEQKLISEEDL.

4. The DNA sequence of HSPA4:

atgtcggtggtgggcatagacctgggcttccagagctgctacgtcgctgtggcccgcgccggcggcatcgagactatcgctaatgagtatagcgaccgctgcacgccggcttgcatttcttttggtcctaagaatcgttcaattggagcagcagctaaaagccaggtaatttctaatgcaaagaacacagtccaaggatttaaaagattccatggccgagcattctctgatccatttgtggaggcagaaaaatctaaccttgcatatgatattgtgcagttgcctacaggattaacaggtataaaggtgacatatatggaggaagagcgaaattttaccactgagcaagtgactgccatgcttttgtccaaactgaaggagacagccgaaagtgttcttaagaagcctgtagttgactgtgttgtttcggttccttgtttctatactgatgcagaaagacgatcagtgatggatgcaacacagattgctggtcttaattgcttgcgattaatgaatgaaaccactgcagttgctcttgcatatggaatctataagcaggatcttcctgccttagaagagaaaccaagaaatgtagtttttgtagacatgggccactctgcttatcaagtttctgtatgtgcatttaatagaggaaaactgaaagttctggccactgcatttgacacgacattgggaggtagaaaatttgatgaagtgttagtaaatcacttctgtgaagaatttgggaagaaatacaagctagacattaagtccaaaatccgtgcattattacgactctctcaggagtgtgagaaactcaagaaattgatgagtgcaaatgcttcagatctccctttgagcattgaatgttttatgaatgatgttgatgtatctggaactatgaatagaggcaaatttctggagatgtgcaatgatctcttagctagagtggagccaccacttcgtagtgttttggaacaaaccaagttaaagaaagaagatatttatgcagtggagatagttggtggtgctacacgaatccctgcggtaaaagagaagatcagcaaatttttcggtaaagaacttagtacaacattaaatgctgatgaagctgtcactcgaggctgtgcattgcagtgtgccatcttatcgcctgctttcaaagtcagagaattttctatcactgatgtagtaccatatccaatatctctgagatggaattctccagctgaagaagggtcaagtgactgtgaagtcttttccaaaaatcatgctgctcctttctctaaagttcttacattttatagaaaggaacctttcactcttgaggcctactacagctctcctcaggatttgccctatccagatcctgctatagctcagttttcagttcagaaagtcactcctcagtctgatggctccagttcaaaagtgaaagtcaaagttcgagtaaatgtccatggcattttcagtgtgtccagtgcatctttagtggaggttcacaagtctgaggaaaatgaggagccaatggaaacagatcagaatgcaaaggaggaagagaagatgcaagtggaccaggaggaaccacatgttgaagagcaacagcagcagacaccagcagaaaataaggcagagtctgaagaaatggagacctctcaagctggatccaaggataaaaagatggaccaaccaccccaagccaagaaggcaaaagtgaagaccagtactgtggacctgccaatcgagaatcagctattatggcagatagacagagagatgctcaacttgtacattgaaaatgagggtaagatgatcatgcaggataaactggagaaggagcggaatgatgctaagaacgcagtggaggaatatgtgtatgaaatgagagacaagcttagtggtgaatatgagaagtttgtgagtgaagatgatcgtaacagttttactttgaaactggaagatactgaaaattggttgtatgaggatggagaagaccagccaaagcaagtttatgttgataagttggctgaattaaaaaatctaggtcaacctattaagatacgtttccaggaatctgaagaacgaccaaaattatttgaagaactagggaaacagatccaacagtatatgaaaataatcagctctttcaaaaacaaggaggaccagtatgatcatttggatgctgctgacatgacaaaggtagaaaaaagcacaaatgaagcaatggagtggatgaataacaagctaaatctgcagaacaagcagagtttgaccatggatccagttgtcaagtcaaaagagattgaagctaaaattaaggagctgacaagtacttgtagccctataatttcaaagcccaaacccaaagtggaacctccaaaagaggaacaaaaaaatgcagagcagaatggaccagtggatggacaaggagacaacccaggcccccaggctgctgagcagggtacagacacagctgtgccttcggattcagacaagaagcttcctgaaatggacattgat.

The amino acid sequence of HSPA4: (840 AAs; 94.33 kDa)

MSVVGIDLGFQSCYVAVARAGGIETIANEYSDRCTPACISFGPKNRSIGAAAKSQVISNAKNTVQGFKRFHGRAFSDPFVEAEKSNLAYDIVQLPTGLTGIKVTYMEEERNFTTEQVTAMLLSKLKETAESVLKKPVVDCVVSVPCFYTDAERRSVMDATQIAGLNCLRLMNETTAVALAYGIYKQDLPALEEKPRNVVFVDMGHSAYQVSVCAFNRGKLKVLATAFDTTLGGRKFDEVLVNHFCEEFGKKYKLDIKSKIRALLRLSQECEKLKKLMSANASDLPLSIECFMNDVDVSGTMNRGKFLEMCNDLLARVEPPLRSVLEQTKLKKEDIYAVEIVGGATRIPAVKEKISKFFGKELSTTLNADEAVTRGCALQCAILSPAFKVREFSITDVVPYPISLRWNSPAEEGSSDCEVFSKNHAAPFSKVLTFYRKEPFTLEAYYSSPQDLPYPDPAIAQFSVQKVTPQSDGSSSKVKVKVRVNVHGIFSVSSASLVEVHKSEENEEPMETDQNAKEEEKMQVDQEEPHVEEQQQQTPAENKAESEEMETSQAGSKDKKMDQPPQAKKAKVKTSTVDLPIENQLLWQIDREMLNLYIENEGKMIMQDKLEKERNDAKNAVEEYVYEMRDKLSGEYEKFVSEDDRNSFTLKLEDTENWLYEDGEDQPKQVYVDKLAELKNLGQPIKIRFQESEERPKLFEELGKQIQQYMKIISSFKNKEDQYDHLDAADMTKVEKSTNEAMEWMNNKLNLQNKQSLTMDPVVKSKEIEAKIKELTSTCSPIISKPKPKVEPPKEEQKNAEQNGPVDGQGDNPGPQAAEQGTDTAVPSDSDKKLPEMDID.

The amino acid sequence fused with His tag (His tag- HSPA4):

HHHHHHMSVVGIDLGFQSCYVAVARAGGIETIANEYSDRCTPACISFGPKNRSIGAAAKSQVISNAKNTVQGFKRFHGRAFSDPFVEAEKSNLAYDIVQLPTGLTGIKVTYMEEERNFTTEQVTAMLLSKLKETAESVLKKPVVDCVVSVPCFYTDAERRSVMDATQIAGLNCLRLMNETTAVALAYGIYKQDLPALEEKPRNVVFVDMGHSAYQVSVCAFNRGKLKVLATAFDTTLGGRKFDEVLVNHFCEEFGKKYKLDIKSKIRALLRLSQECEKLKKLMSANASDLPLSIECFMNDVDVSGTMNRGKFLEMCNDLLARVEPPLRSVLEQTKLKKEDIYAVEIVGGATRIPAVKEKISKFFGKELSTTLNADEAVTRGCALQCAILSPAFKVREFSITDVVPYPISLRWNSPAEEGSSDCEVFSKNHAAPFSKVLTFYRKEPFTLEAYYSSPQDLPYPDPAIAQFSVQKVTPQSDGSSSKVKVKVRVNVHGIFSVSSASLVEVHKSEENEEPMETDQNAKEEEKMQVDQEEPHVEEQQQQTPAENKAESEEMETSQAGSKDKKMDQPPQAKKAKVKTSTVDLPIENQLLWQIDREMLNLYIENEGKMIMQDKLEKERNDAKNAVEEYVYEMRDKLSGEYEKFVSEDDRNSFTLKLEDTENWLYEDGEDQPKQVYVDKLAELKNLGQPIKIRFQESEERPKLFEELGKQIQQYMKIISSFKNKEDQYDHLDAADMTKVEKSTNEAMEWMNNKLNLQNKQSLTMDPVVKSKEIEAKIKELTSTCSPIISKPKPKVEPPKEEQKNAEQNGPVDGQGDNPGPQAAEQGTDTAVPSDSDKKLPEMDID.

5. The DNA sequence of ENO1:

atgtctattctcaagatccatgccagggagatctttgactctcgcgggaatcccactgttgaggttgatctcttcacctcaaaaggtctcttcagagctgctgtgcccagtggtgcttcaactggtatctatgaggccctagagctccgggacaatgataagactcgctatatggggaagggtgtctcaaaggctgttgagcacatcaataaaactattgcgcctgccctggttagcaagaaactgaacgtcacagaacaagagaagattgacaaactgatgatcgagatggatggaacagaaaataaatctaagtttggtgcgaacgccattctgggggtgtcccttgccgtctgcaaagctggtgccgttgagaagggggtccccctgtaccgccacatcgctgacttggctggcaactctgaagtcatcctgccagtcccggcgttcaatgtcatcaatggcggttctcatgctggcaacaagctggccatgcaggagttcatgatcctcccagtcggtgcagcaaacttcagggaagccatgcgcattggagcagaggtttaccacaacctgaagaatgtcatcaaggagaaatatgggaaagatgccaccaatgtgggggatgaaggcgggtttgctcccaacatcctggagaataaagaaggcctggagctgctgaagactgctattgggaaagctggctacactgataaggtggtcatcggcatggacgtagcggcctccgagttcttcaggtctgggaagtatgacctggacttcaagtctcccgatgaccccagcaggtacatctcgcctgaccagctggctgacctgtacaagtccttcatcaaggactacccagtggtgtctatcgaagatccctttgaccaggatgactggggagcttggcagaagttcacagccagtgcaggaatccaggtagtgggggatgatctcacagtgaccaacccaaagaggatcgccaaggccgtgaacgagaagtcctgcaactgcctcctgctcaaagtcaaccagattggctccgtgaccgagtctcttcaggcgtgcaagctggcccaggccaatggttggggcgtcatggtgtctcatcgttcgggggagactgaagataccttcatcgctgacctggttgtggggctgtgcactgggcagatcaagactggtgccccttgccgatctgagcgcttggccaagtacaaccagctcctcagaattgaagaggagctgggcagcaaggctaagtttgccggcaggaacttcagaaaccccttggccaag.

The amino acid sequence of ENO1: (434 AAs; 47.17 kDa)

MSILKIHAREIFDSRGNPTVEVDLFTSKGLFRAAVPSGASTGIYEALELRDNDKTRYMGKGVSKAVEHINKTIAPALVSKKLNVTEQEKIDKLMIEMDGTENKSKFGANAILGVSLAVCKAGAVEKGVPLYRHIADLAGNSEVILPVPAFNVINGGSHAGNKLAMQEFMILPVGAANFREAMRIGAEVYHNLKNVIKEKYGKDATNVGDEGGFAPNILENKEGLELLKTAIGKAGYTDKVVIGMDVAASEFFRSGKYDLDFKSPDDPSRYISPDQLADLYKSFIKDYPVVSIEDPFDQDDWGAWQKFTASAGIQVVGDDLTVTNPKRIAKAVNEKSCNCLLLKVNQIGSVTESLQACKLAQANGWGVMVSHRSGETEDTFIADLVVGLCTGQIKTGAPCRSERLAKYNQLLRIEEELGSKAKFAGRNFRNPLAK.

The amino acid sequence fused with His tag (ENO1-His tag):

MSILKIHAREIFDSRGNPTVEVDLFTSKGLFRAAVPSGASTGIYEALELRDNDKTRYMGKGVSKAVEHINKTIAPALVSKKLNVTEQEKIDKLMIEMDGTENKSKFGANAILGVSLAVCKAGAVEKGVPLYRHIADLAGNSEVILPVPAFNVINGGSHAGNKLAMQEFMILPVGAANFREAMRIGAEVYHNLKNVIKEKYGKDATNVGDEGGFAPNILENKEGLELLKTAIGKAGYTDKVVIGMDVAASEFFRSGKYDLDFKSPDDPSRYISPDQLADLYKSFIKDYPVVSIEDPFDQDDWGAWQKFTASAGIQVVGDDLTVTNPKRIAKAVNEKSCNCLLLKVNQIGSVTESLQACKLAQANGWGVMVSHRSGETEDTFIADLVVGLCTGQIKTGAPCRSERLAKYNQLLRIEEELGSKAKFAGRNFRNPLAKHHHHHH.

6. The DNA sequence of EIF5A:

ATGGCAGATGACTTGGACTTCGAGACAGGAGATGCAGGGGCCTCAGCCACCTTCCCAATGCAGTGCTCAGCATTACGTAAGAATGGCTTTGTGGTGCTCAAAGGCCGGCCATGTAAGATCGTCGAGATGTCTACTTCGAAGACTGGCAAGCACGGCCACGCCAAGGTCCATCTGGTTGGTATTGACATCTTTACTGGGAAGAAATATGAAGATATCTGCCCGTCAACTCATAATATGGATGTCCCCAACATCAAAAGGAATGACTTCCAGCTGATTGGCATCCAGGATGGGTACCTATCACTGCTCCAGGACAGCGGGGAGGTACGAGAGGACCTTCGTCTCCCTGAGGGAGACCTTGGCAAGGAGATTGAGCAGAAGTACGACTGTGGAGAAGAGATCCTGATCACGGTGCTGTCTGCCATGACAGAGGAGGCAGCTGTTGCAATCAAGGCCATGGCAAAA.

The amino acid sequence of EIF5A: (154 AAs; 16.83 kDa)

MADDLDFETGDAGASATFPMQCSALRKNGFVVLKGRPCKIVEMSTSKTGKHGHAKVHLVGIDIFTGKKYEDICPSTHNMDVPNIKRNDFQLIGIQDGYLSLLQDSGEVREDLRLPEGDLGKEIEQKYDCGEEILITVLSAMTEEAAVAIKAMAK.

The amino acid sequence fused with His tag (EIF5A-His tag):

MADDLDFETGDAGASATFPMQCSALRKNGFVVLKGRPCKIVEMSTSKTGKHGHAKVHLVGIDIFTGKKYEDICPSTHNMDVPNIKRNDFQLIGIQDGYLSLLQDSGEVREDLRLPEGDLGKEIEQKYDCGEEILITVLSAMTEEAAVAIKAMAKHHHHHH.

Protein labeling and enrichment. Lysates from tumor and paracancerous breast tissues were incubated with IR-780-alkyne under optimized conditions to enable selective covalent labeling of tumor signature proteins. After covalent labeling, biotin was conjugated to the dye-labeled proteins *via* a copper-catalyzed azide-alkyne cycloaddition (click reaction). The reaction mixtures were processed through a series of organic solvent precipitation and washing steps to remove impurities. The labeled proteins were then incubated with streptavidin agarose resin (20512ES08, Yeasen) under gentle agitation to enable streptavidin-biotin affinity capture. After thorough washing, the enriched proteins were eluted from the resin by heat treatment in detergent-containing buffer. The resulting purified proteins were used for downstream SDS-PAGE and mass spectrometry analysis.

Proteomics technique. To identify the covalent modification sites of IR-780-alkyne on a representative tumor signature protein, the FUS protein standard was first mixed with IR-780-alkyne and incubated at 60^o^C for 2 h to allow the protein-dye reaction to occur. The resulting samples were then subjected to proteomics analysis. Both reduced and non-reduced forms of the protein samples were prepared. After alkylation with iodoacetamide, the proteins were separately digested with trypsin and chymotrypsin under optimized conditions. The resulting peptides were desalted and analyzed using a high-resolution Orbitrap Fusion Lumos mass spectrometer coupled with high-performance liquid chromatography-mass spectrometry (HPLC-MS). Mass spectra were acquired in data-dependent acquisition (DDA) mode with a full MS scan range of m/z 300-1800, followed by MS/MS scans. The data were processed using BioPharma Finder software for peptide mapping, with variable modifications including oxidation, deamidation, carbamidomethylation, and custom-defined IR-780-alkyne adducts. Modification sites were identified with high confidence and mapped to the corresponding cysteine residues.

Statistical analyses. Data points were organized in Microsoft Excel, and statistical analyses were carried out using GraphPad Prism and OriginPro. Statistical differences were assessed with a two-tailed Student’s t-test, considering p < 0.05 as significant. Continuous variables were expressed as mean ± standard deviation. The similarity between the tumor profile predicted by the NIR-FPs-based detection system and the corresponding pathology-confirmed tumor profile was calculated in MATLAB using the two contour images as inputs. After grayscale conversion, binarization, area normalization, and Frobenius norm normalization, the maximum matching ratio obtained by convolution-based matching was used as the final similarity value.

# S2 Supplementary Tables

**Table S1.** Clinical and histopathological information of the tumors in our patient cohort.

| Patient ID | Tumor size [cm] | Stage at diagnosis | Molecular classification |
| --- | --- | --- | --- |
| R0115 | 2×1.5×1 | T2/N1 | luminal B |
| R0178 | 2×1.8×1.5 | T1c/N2 | luminal A |
| R0214 | 2×1.5×1.5 | T1c/N1 | luminal B |
| R0222 | 2×2×1.8 | T1c/N0 | Basal like |
| R0274 | 5×5×2 | T2/N0 | Basal like |
| R0176 | 2.5×2×1.8 | T2/N1 | Basal like |
| R0235 | 1.8×1.5×1.2 | T1c/N0 | luminal A |
| R0331 | 2.7×2.5×1.9 | - | luminal A |
| R0573 | 2.5×2.3×2 | T2/N1 | Basal like |
| R0599 | 1.8×1×0.8 | T1c/N0 | luminal B |
| R0189 | 1.3×0.7×0.7 | T1c/N0 | luminal B |
| R0192 | 1.8×1.5×1.5 | T1c/N0 | luminal B |
| R0211 | 4.5×3×2 | T1a/N0 | luminal A |
| R0225 | 6×4×1.5 | T3/N1 | luminal B |
| R0226 | 2×1.8×1.5 | T1c/N0 | luminal A |
| R0227 | 3.5×3×2.5 | T2/N1 | luminal B |
| R0232 | 3×2.5×2 | T2/N0 | Basal like |
| R0236 | 2.2×2×1.5 | T2/N0 | luminal A |
| R0244 | 2.5×2×1.3 | T2/N1 | luminal A |
| R0252 | 4.5×4×3.5 | T2/N1 | luminal B |
| R0281 | 3.5×2.5×2 | T2/N0 | luminal B |
| R0282 | 3.5×2×1.3 | T2/N2 | luminal A |
| R0298 | 5×5×3.5 | T2/N2 | luminal A |
| R0304 | 3.5×2.5×2 | T2/N0 | luminal A |
| R0315 | 3×2.5×1.5 | - | luminal A |
| R0328 | 1.8×1.5×1.2 | T1c/N1 | luminal A |
| R0332 | 2.5×1.5×1.2 | T2/N1 | luminal A |
| R0338 | 2.5×1.5×1 | T1c/N1 | luminal A |
| R0418 | 3×2×1.6 | T2/N1 | luminal A |
| R0429 | 5.5×4×2.5 | T3/N1 | luminal B |
| R0445 | 3.5×3×2.8 | T2/N0 | luminal B |
| R0491 | 2.2×2×1.5 | T2/N1 | luminal A |
| R0510 | 5.1×1.2×2.5 | T3/N1 | - |
| R0667 | 5.5×4.5×2.8 | T3/N0 | luminal A |
| RC001 | - | - | luminal B |
| RC004 | - | - | TNBC |
| RC007 | - | - | HER2+ |

# S3 Supplementary Figures





**Fig. S1. (a)** Chemical structures of tumor-selective dyes. **(b)** Normalized UV-absorption spectroscopy and fluorescence spectroscopy of the IR-6B1, IR-6B3, IR-3B3, IR-780-alkyne, IR-6B3C, IR-6B3S, IR-6B4S, and IR-6B6C.

**
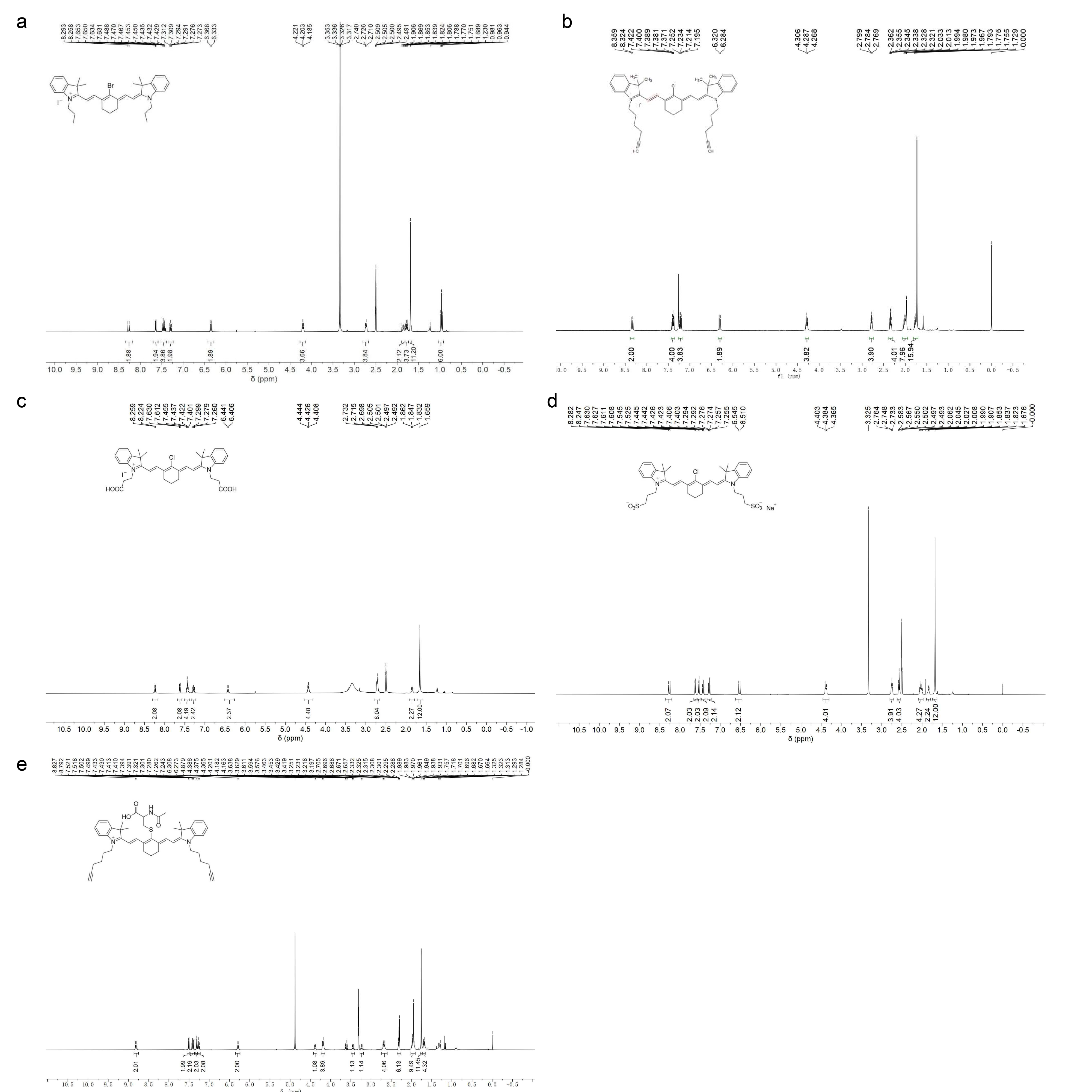
**

**Fig. S2.** ^1^H-NMR spectra of **(a)** IR-3B3, **(b)** IR-780-alkyne, **(c)** IR-6B3C, **(d)** IR-6B3S, and **(e)** IR-780-alkyne-AC.

**
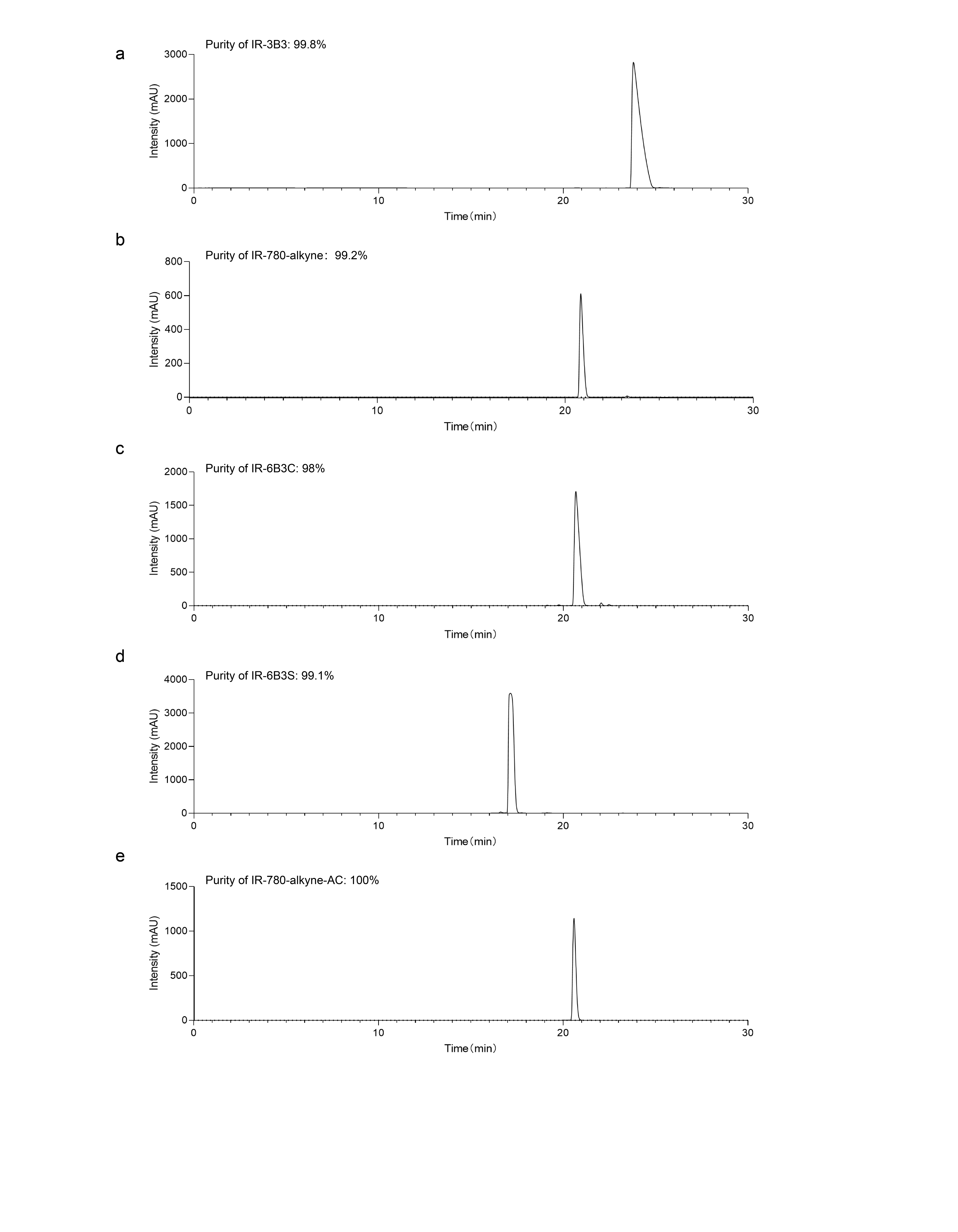
**

**Fig. S3.** Analytical HPLC chromatograms of **(a)** IR-3B3, **(b)** IR-780-alkyne, **(c)** IR-6B3C, **(d)** IR-6B3S, and **(e)** IR-780-alkyne-AC, showing purities of 99.8%, 99.2%, 98%, 99.1%, and 100%, respectively.

**
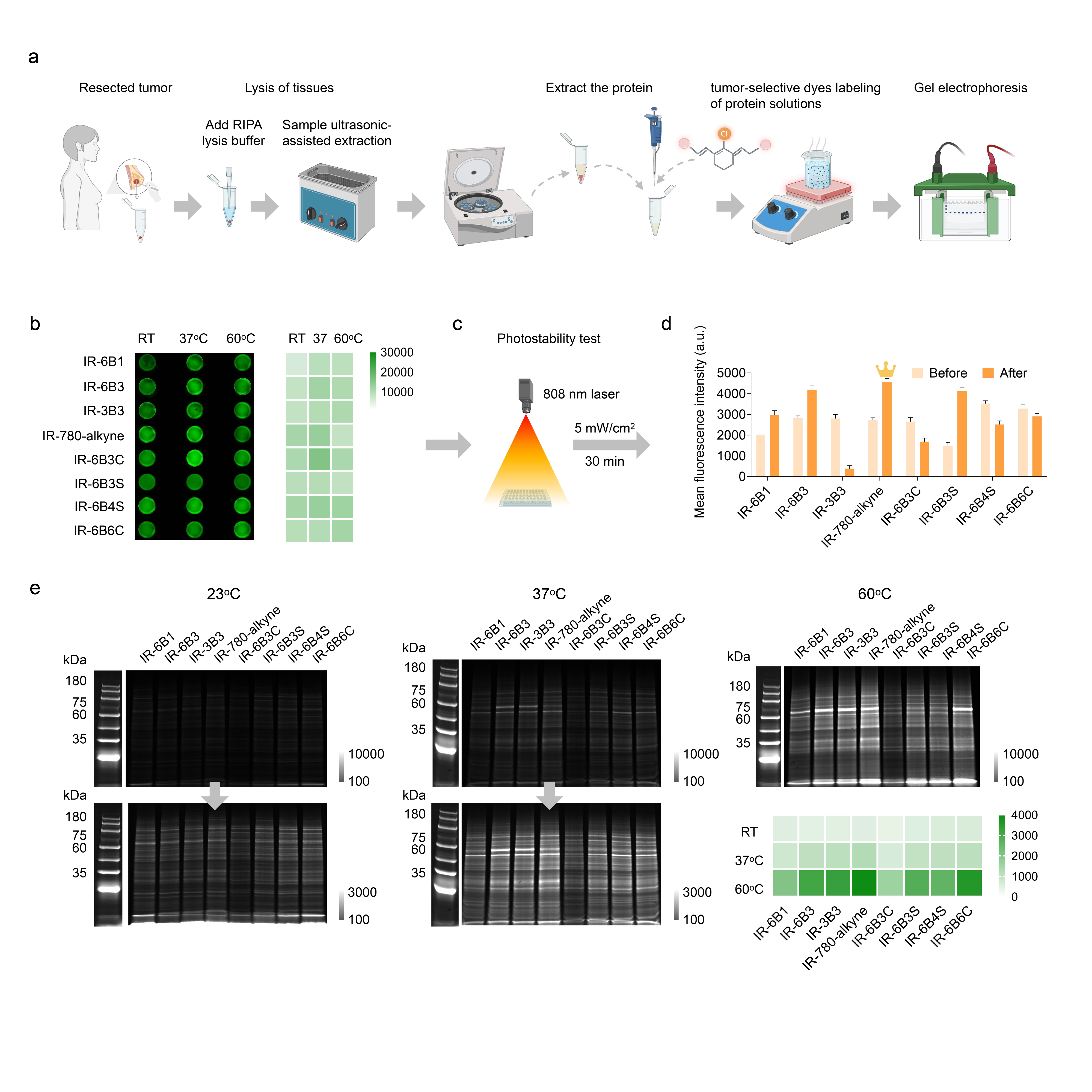
**

**Fig. S4. (a)** The flow chart of SDS-PAGE gel electrophoresis images of the tumor-selective dyes labeling of breast cancer lysate. **(b)** Fluorescence images and intensity heat map of tumor-selective dyes in breast cancer lysate at various reaction temperatures. The concentration of lysate@dyes (PBS) was 0.2 µmol. **(c)** Setup for photostability testing of dye-labeled tumor signature proteins under 808 nm laser exposure (5 mW/cm², 30 min). **(d)** Fluorescence intensity of the dye solutions in microplates before and after the 808 nm laser excitation. **(e)** Fluorescence scanning images and heat map statistics of protein bands after the eight dyes incubated with human breast tumor lysate at 23^o^C, 37^o^C and 60^o^C. (The figure below is adjusted to their respective optimal scale). Schemes were created with BioRender.com.





**Fig. S5.** Optical characterization and ambient-light photostability of the HSA@IR-780-alkyne model NIR-FP system. **(a)** Absorption spectrum of HSA@IR-780-alkyne, showing a maximum at 787 nm. **(b)** Fluorescence emission spectrum of HSA@IR-780-alkyne, showing a maximum at 822 nm. **(c)** Representative fluorescence images and quantitative fluorescence intensity of HSA@IR-780-alkyne under ambient-light exposure from 0 h to 12 h, showing favorable fluorescence stability over time.


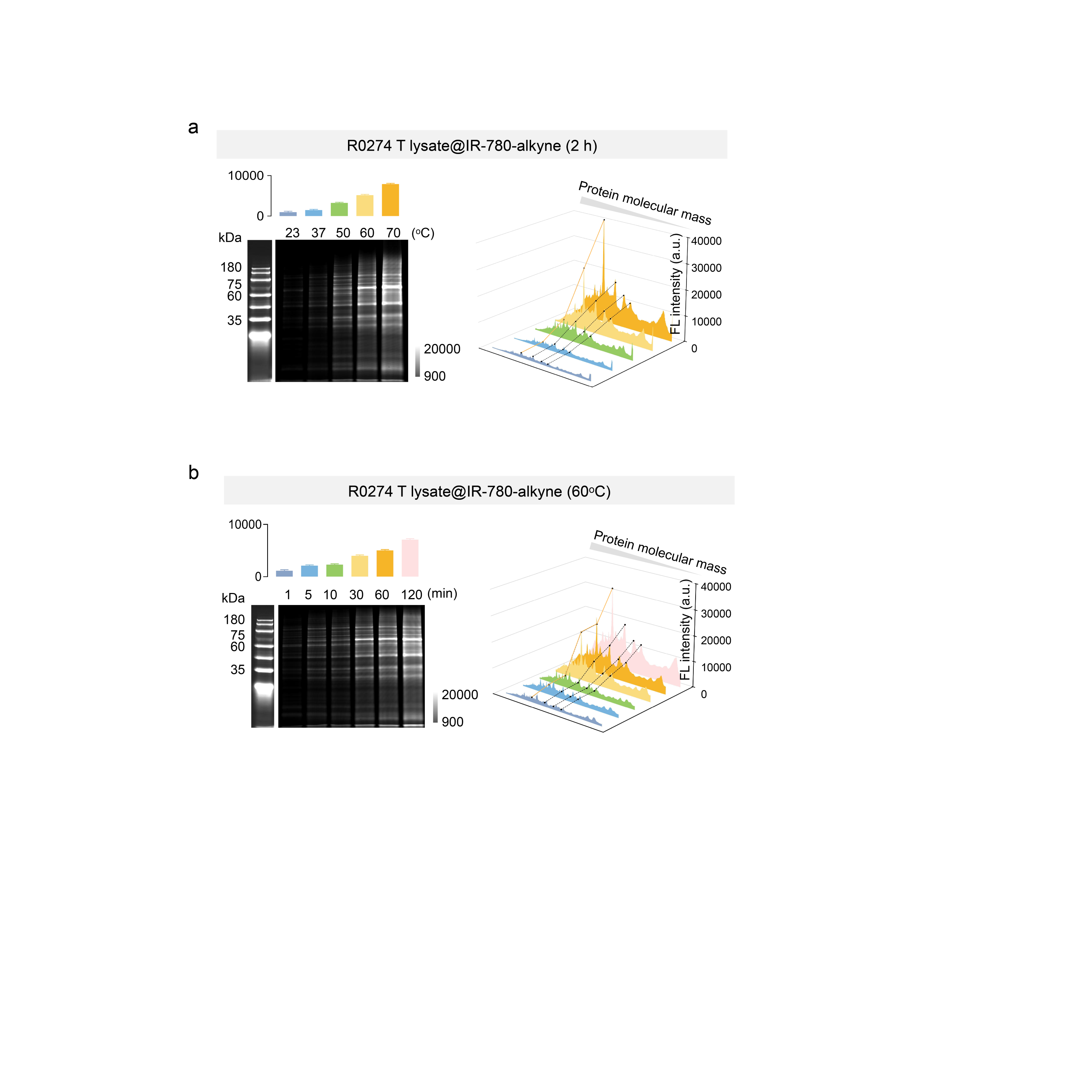


**Fig. S6. (a)** SDS-PAGE gel electrophoresis image and fluorescence intensity analysis of protein bands of dye incubated with human breast tumor lysate (R0274) at various reaction temperatures (23^o^C, 37^o^C, 50^o^C, 60^o^C and 70^o^C) for 2 h. **(b)** SDS-PAGE gel electrophoresis image and fluorescence intensity analysis of protein bands of dye incubated with human breast tumor lysate (R0274) at 60^o^C for different reaction times. (1 min, 5 min, 10 min, 30 min, 60 min, and 120 min).

**
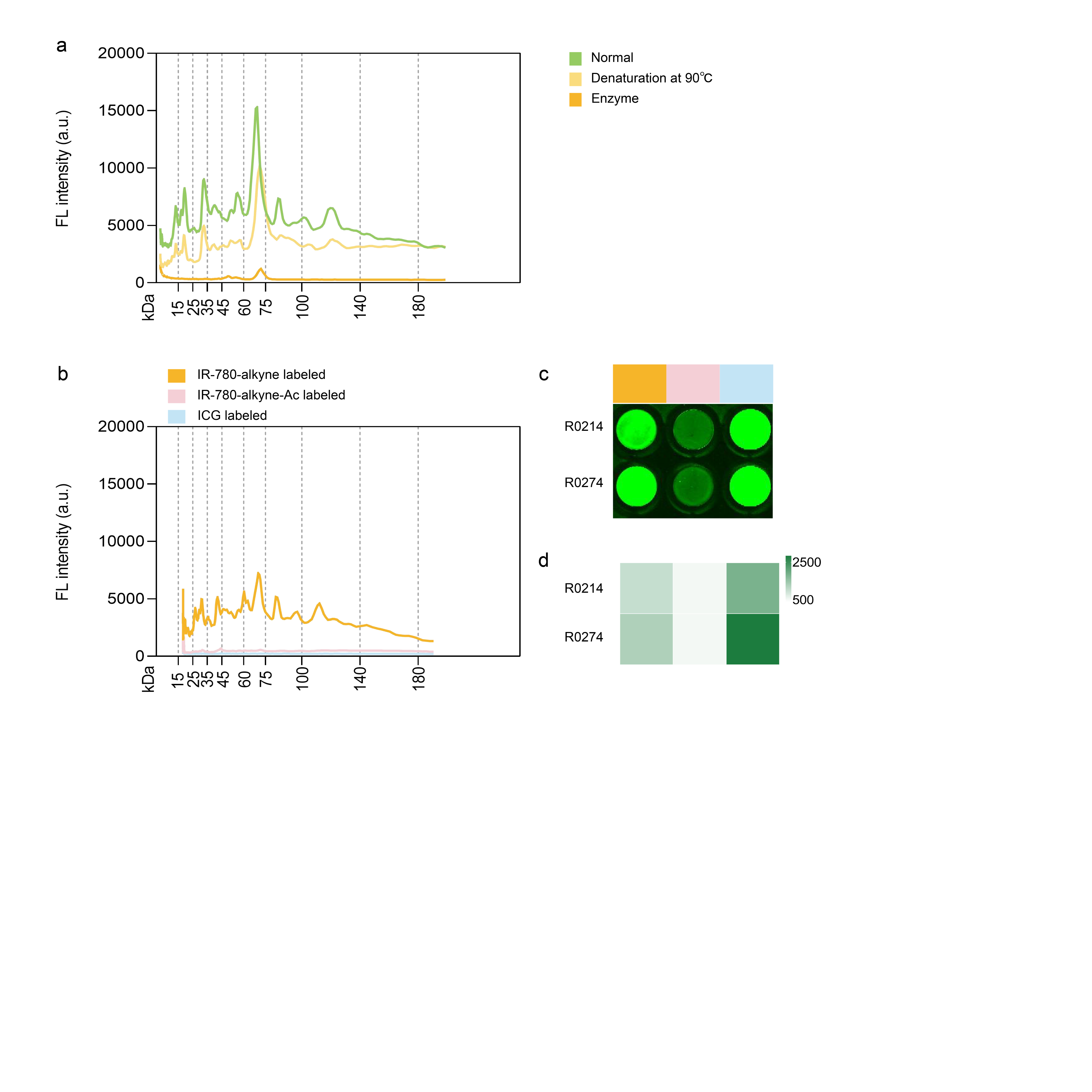
**

**Fig. S7. (a)** Fluorescence analysis of breast tumor lysates under different conditions: normal, heated at 90^o^C for 30 min, or digested with trypsin, followed by incubation with IR-780-alkyne at 60^o^C for 2 h. (Fig. 1d). **(b)** The fluorescence intensity analysis of Fig. 1d. **(c)** The brightness of the mixed solution in the orifice plate and **(d)** the signal quantification in the heat map of IR-780-alkyne, IR-780-alkyne-AC and ICG incubated with breast tumor lysates at 2 h at 60^o^C.





**Fig. S8. (a)** SDS-PAGE gel electrophoresis image of protein bands of human breast tumor lysate (R0214) incubated with dye (IR-780-alkyne, IR-780-alkyne-AC and ICG) at 60^o^C for 2 h. **(b)** SDS-PAGE gel electrophoresis image and **(c)** fluorescence intensity analysis in the range of three molecular weights of human breast tumor lysate (R0274) protein bands incubated with different concentrations (5 μmol, 10 μmol, 20 μmol, 40 μmol and 80 μmol) of dye for 2 h at 60^o^C.

**
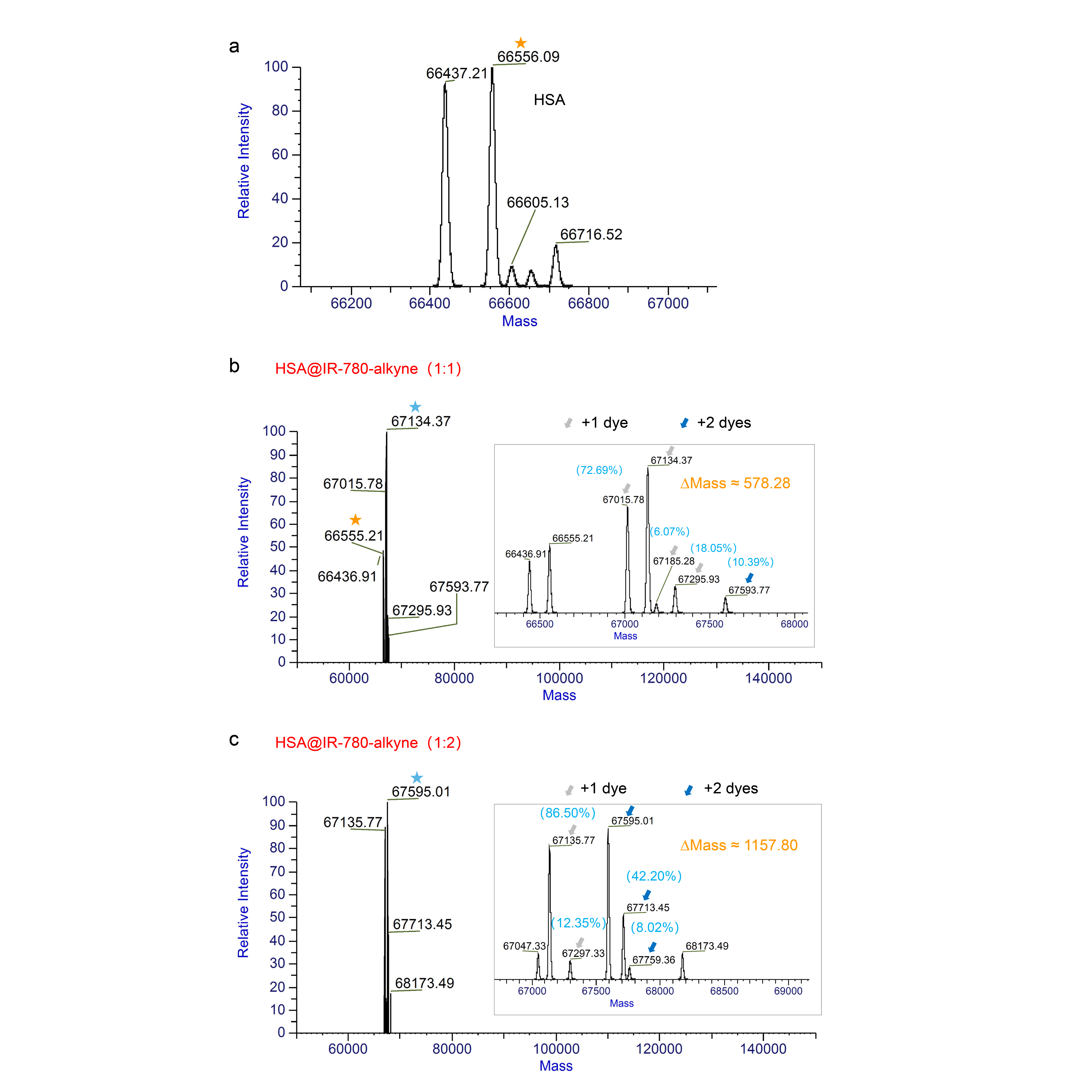
**

**Fig. S9.** High-resolution mass spectrometry of **(a)** HSA **(b)** HSA@IR-780-alkyne (1:1) and **(c)** HSA@IR-780-alkyne (1:2).

**
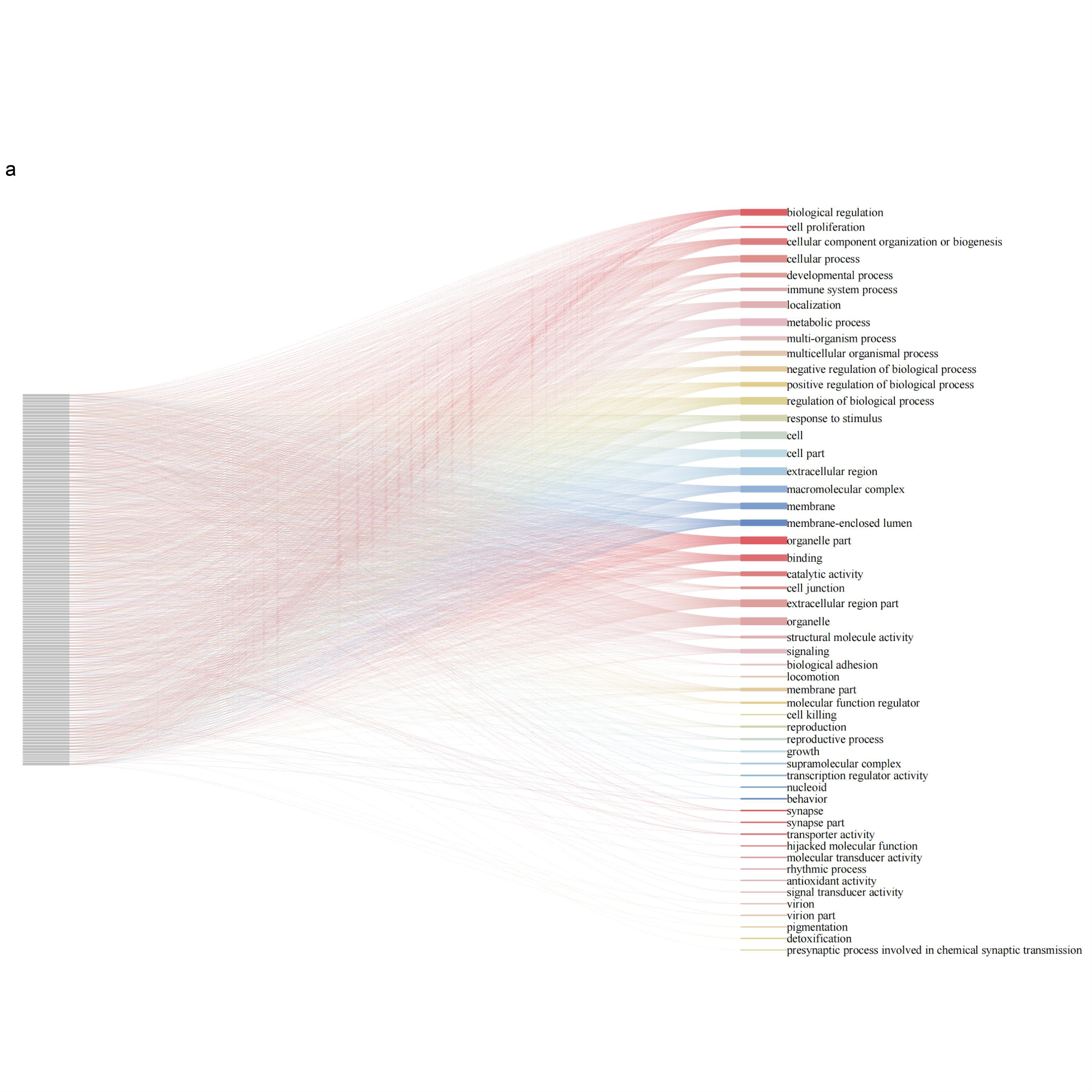
**

**Fig. S10. (a)** Gene Ontology (GO) enrichment analysis of 134 differentially enriched proteins identified by LC-MS from IR-780-alkyne-labeled tumor tissues. Functional annotation spans biological processes, cellular components, and molecular functions, highlighting their roles in cell proliferation, immune regulation, signaling, and structural organization.

**
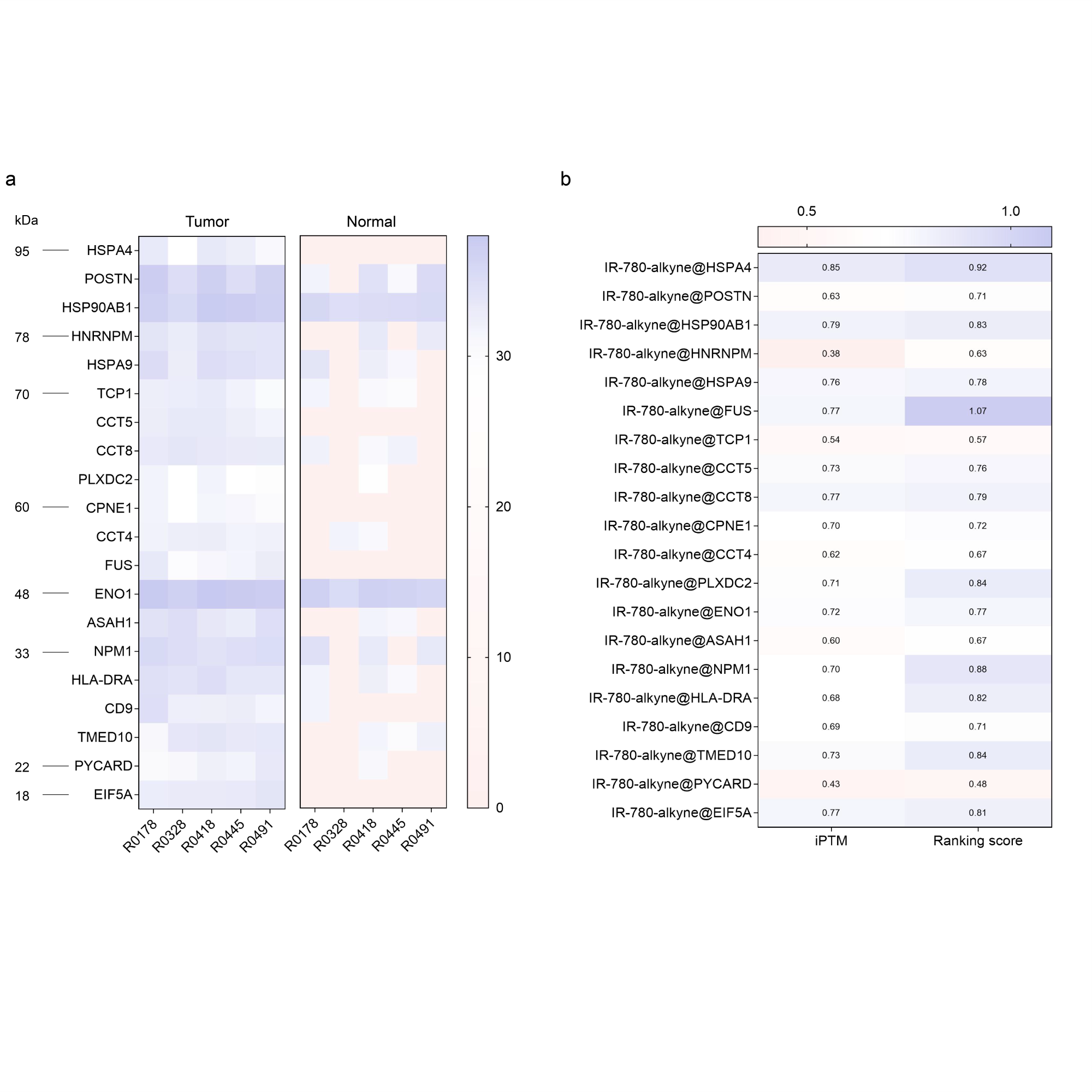
Fig. S11. (a)** Heatmap showing the distribution of 20 tumor-related signature proteins in tumor tissues and normal tissues from five breast cancer patients. **(b)** Binding predictions between IR-780-alkyne and the 20 tumor-related signature proteins based on AlphaFold3 structural modeling. Interaction potential was evaluated by predicted template modeling (iPTM) scores and overall binding ranking scores.

**
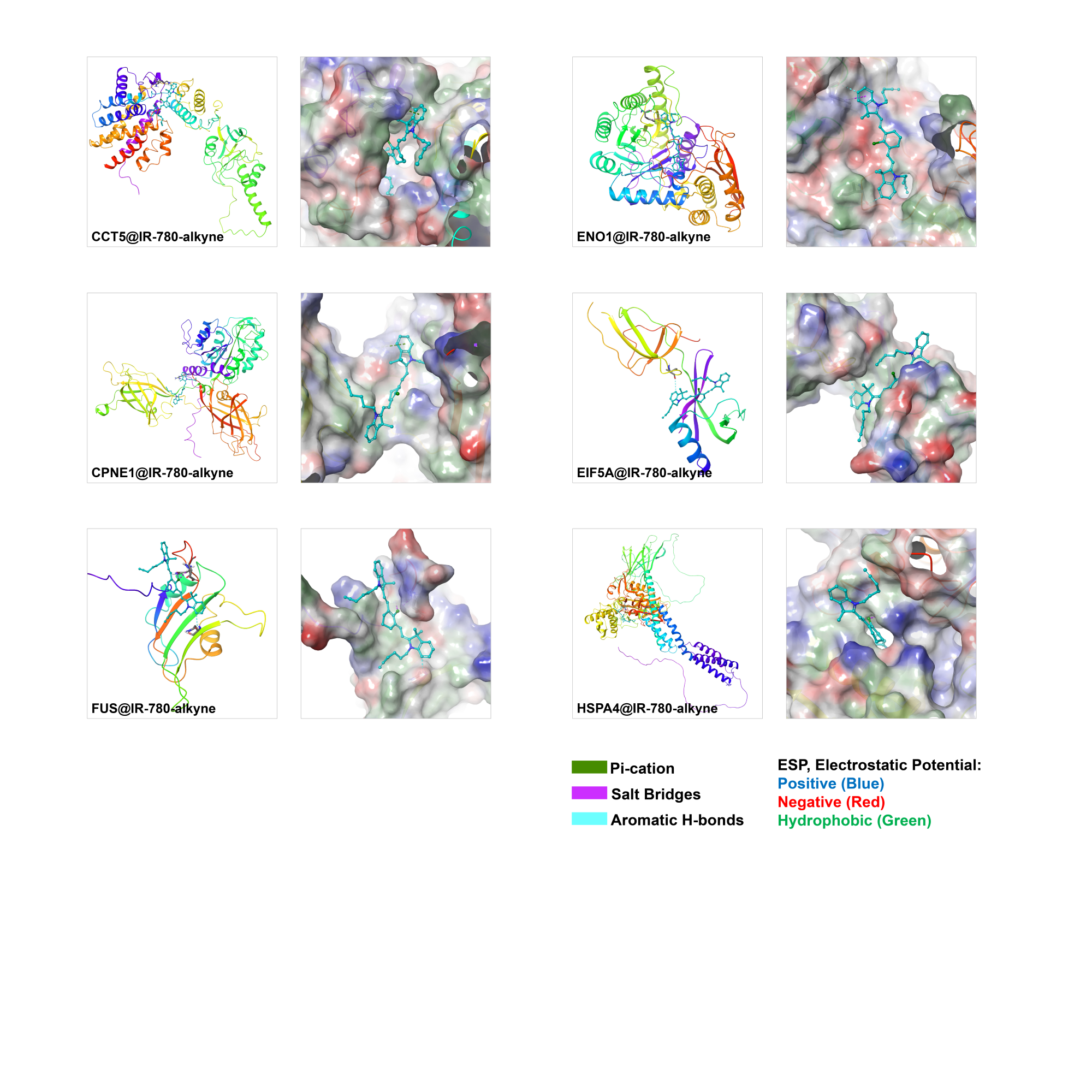
**

**Fig. S12.** Global and local 3D plots of molecular docking simulations by glide program-based non-covalent molecular docking simulation between IR-780-alkyne and six biomarkers (CCT5, ENO1, CPNE1, EIF5A, FUS, and HSPA4).

**
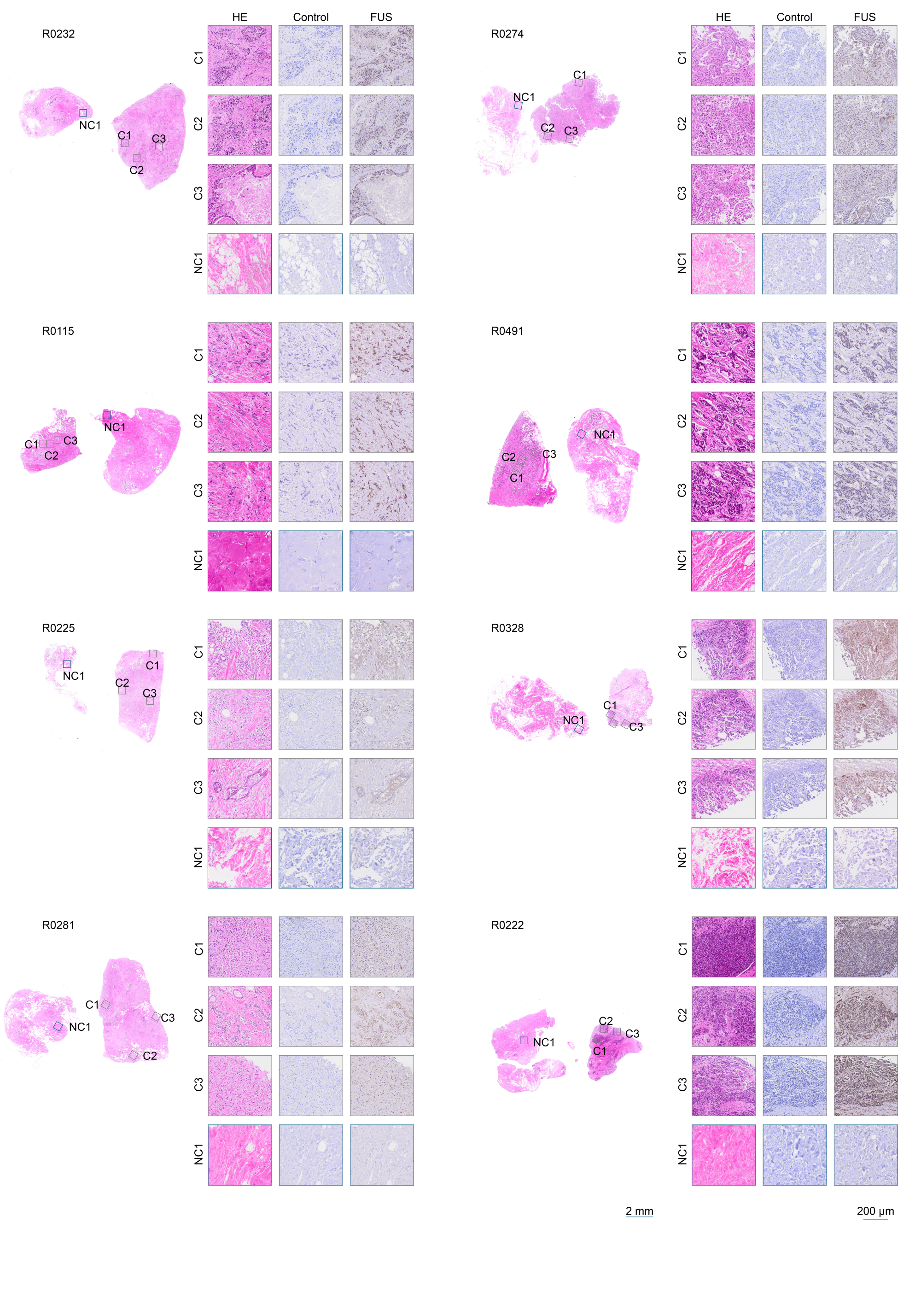
**

**Fig. S13.** Histological and immunohistochemical analysis of breast tumor tissue. H&E staining, negative control, and IHC staining for FUS were performed on adjacent sections of the same tumor region.





**Fig. S14.** Total ion flow chromatograms after **(a)** trypsin and **(b)** chymotrypsin enzyme digestion of FUS@IR-780-alkyne.


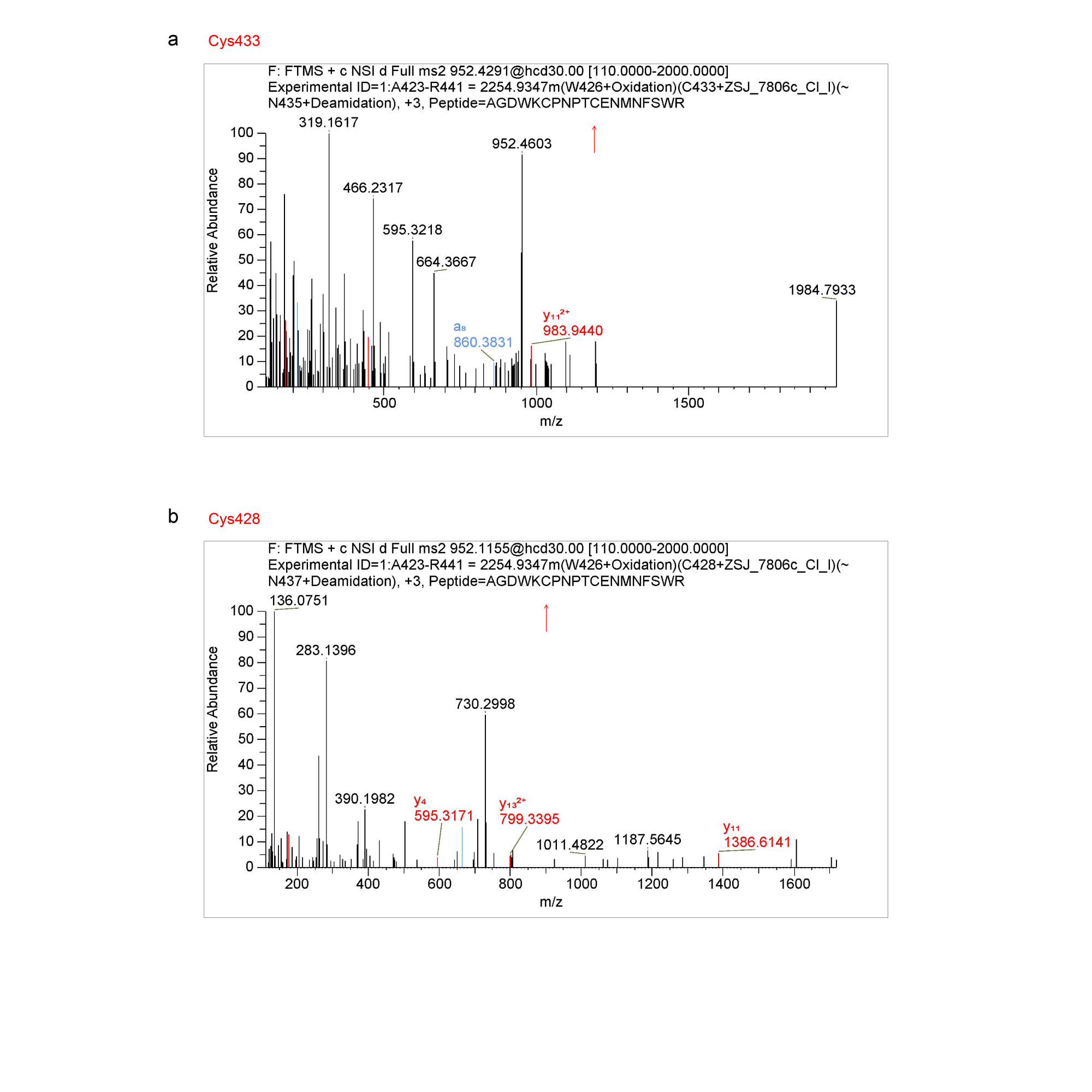


**Fig. S15.** The secondary mass spectra of the sequence of AGDWKCPNPTCENMNFSWR: **(a)** Cys433 and **(b)** Cys428.


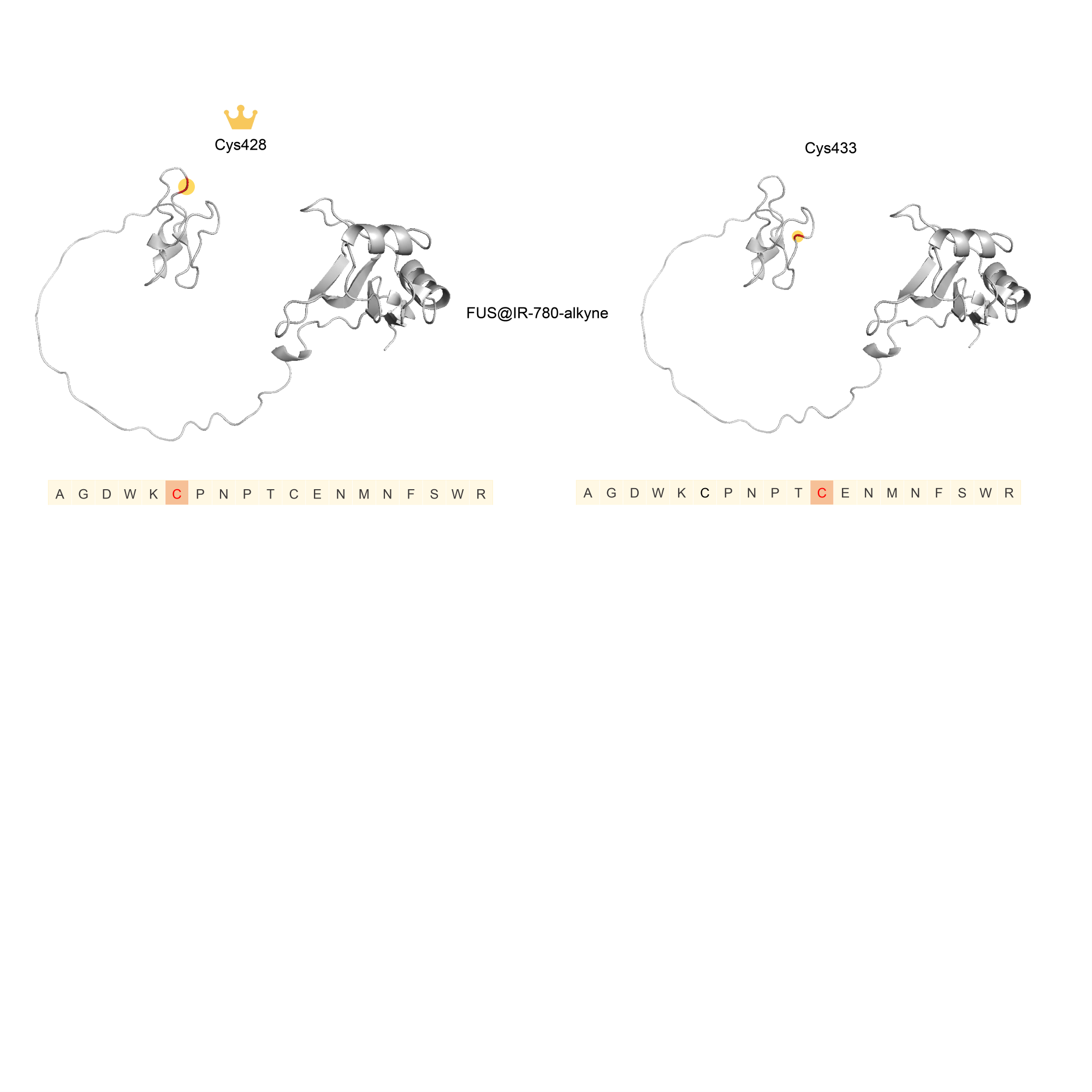


**Fig. S16.** Distribution of cysteine sites recognized by proteomics analysis.

**

**

**Fig. S17. (a)** SDS-PAGE gel electrophoresis image and **(b)** Fluorescence intensity analysis of protein bands of human breast paracancer lysate (R0274) incubated with dye (IR-780-alkyne) at various temperatures (23^o^C, 37^o^C, 50^o^C, 60^o^C and 70^o^C) for 2 h. **(c)** Fluorescence intensity of protein bands after the dye was mixed with lysates of breast cancer and paracancerous tissues at various temperatures for 2 h. **(d)** SDS-PAGE gel electrophoresis image and **(e)** Fluorescence intensity analysis of protein bands of human breast paracancer lysate (R0274) incubated with dye (IR-780-alkyne) at 60^o^C for different reaction times (1 min, 5 min, 10 min, 30 min, 1 h and 2 h). **(f)** Fluorescence intensity of protein bands after the dye was mixed with lysates of breast cancer and paracancerous tissues at 60^o^C for various times. The tumor/paracancerous tissue ratio of R0274 at **(g)** various temperatures for 2 h and **(h)** 60^o^C for different reaction times. **(i)** Comparison of tumor/paracancerous tissue ratios labeled with IR-780 and IR-780-alkyne at different temperatures.

**
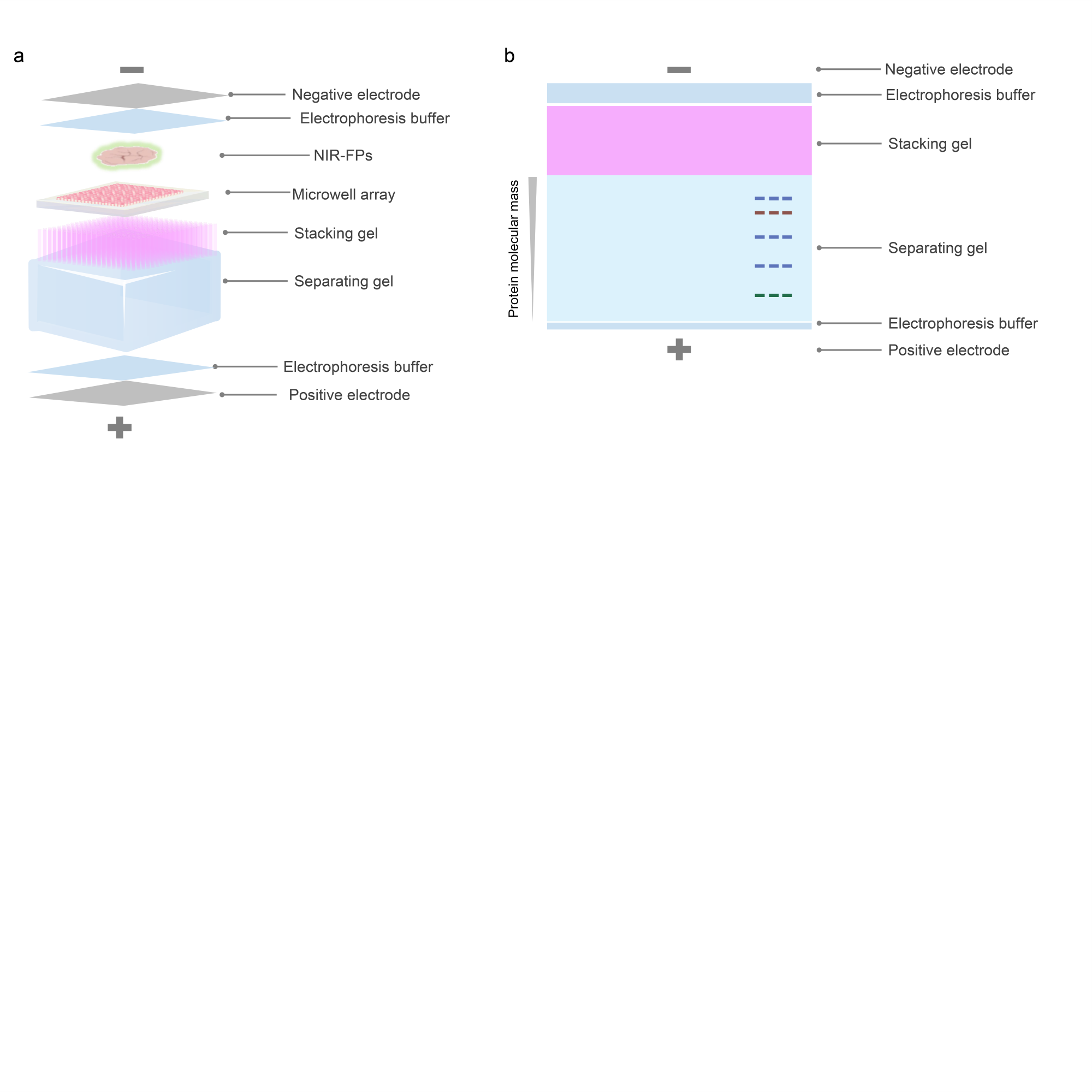
**

**Fig. S18. (a)** Schematic diagram of the 3D histological electrophoresis device integrated into the NIR-FPs-based detection system, comprising a negative electrode, a stacking gel with microwell array, a separating gel, and a positive electrode. **(b)** Illustration of the 3D histological electrophoresis procedure obtained after separation of protein standards (protein marker). Schemes were created with BioRender.com.





**Fig. S19.** Fluorescence images and signal quantification of the eight fractionated layers for the tumor&paracancerous samples **(a)** R0274, **(b)** R0573 and **(c)** R0599 after incubation with IR-780 and IR-780-alkyne following the 3D histological electrophoresis. (Reaction at 23^o^C for 30 min). The tumor/paracancerous tissue ratio of the samples **(d)** R0274, **(e)** R0573 and **(f)** R0599.

**
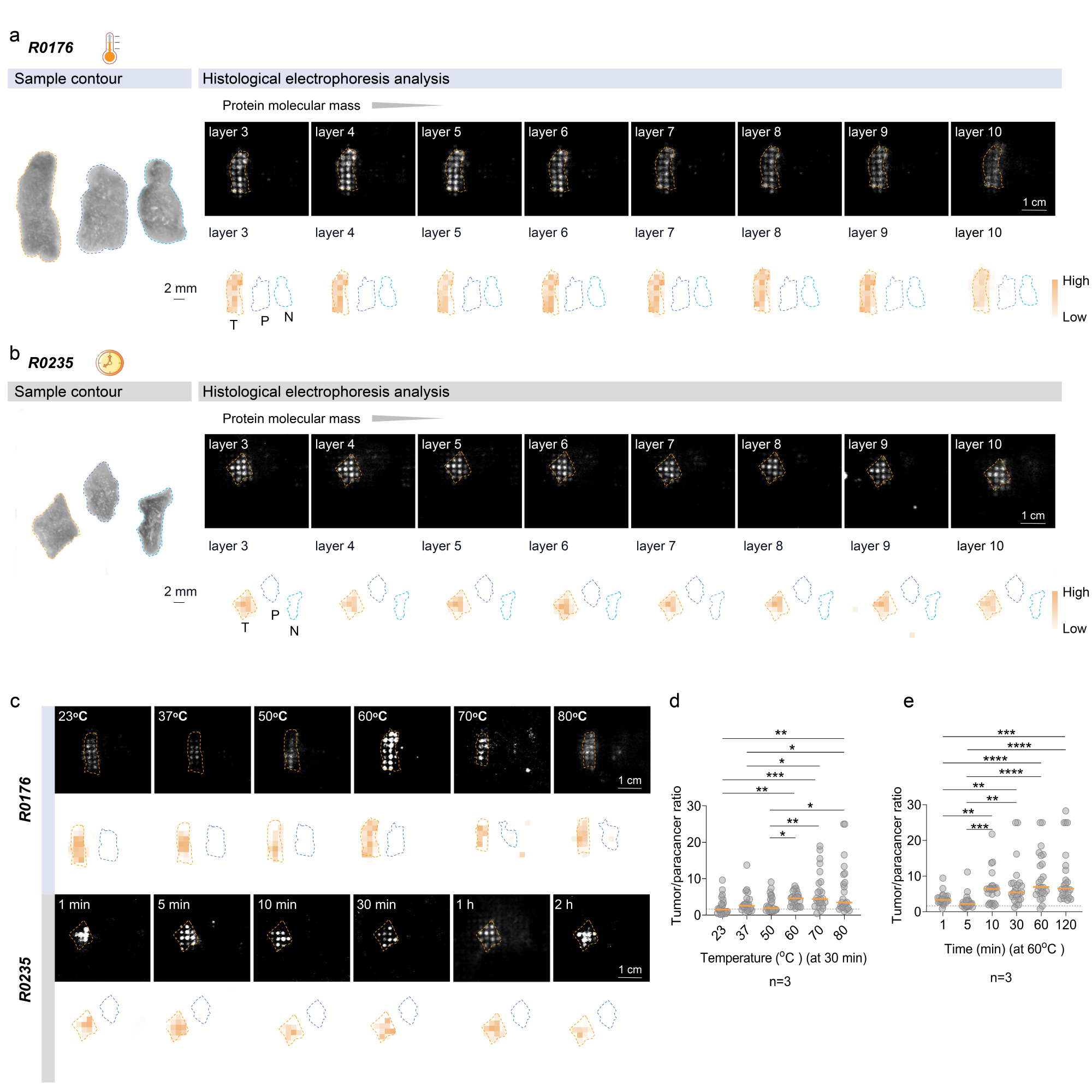
Fig. S20. (a** and **b)** Representative images of breast cancer samples (R0176 and R0235), together with fluorescence images and signal quantification of the eight fractionated layers from the corresponding tumor and paracancerous samples following the NIR-FPs-based detection system. **(c)** Fluorescence images and signal quantification of the one fractionated layer for the tumor&paracancerous samples (R0176 and R0235) following the NIR-FPs-based detection system at various reaction temperatures and times. **(d)** The calculated tumor to paracancerous tissue (n=3, R0176/R0331/R0599) ratios (n = 8 for each breast cancer sample) at various reaction temperatures for 30 min. **(e)** The calculated tumor-to-paracancerous tissue (n=3, R0274/R0176/R0235, other results were analyzed from Fig.S21) ratios (n = 8 for each breast cancer sample) at 60^o^C for various reaction times. Data note: The tumor/paracancerous tissue ratio values exceeding 15 were artificially plotted as 15.

**
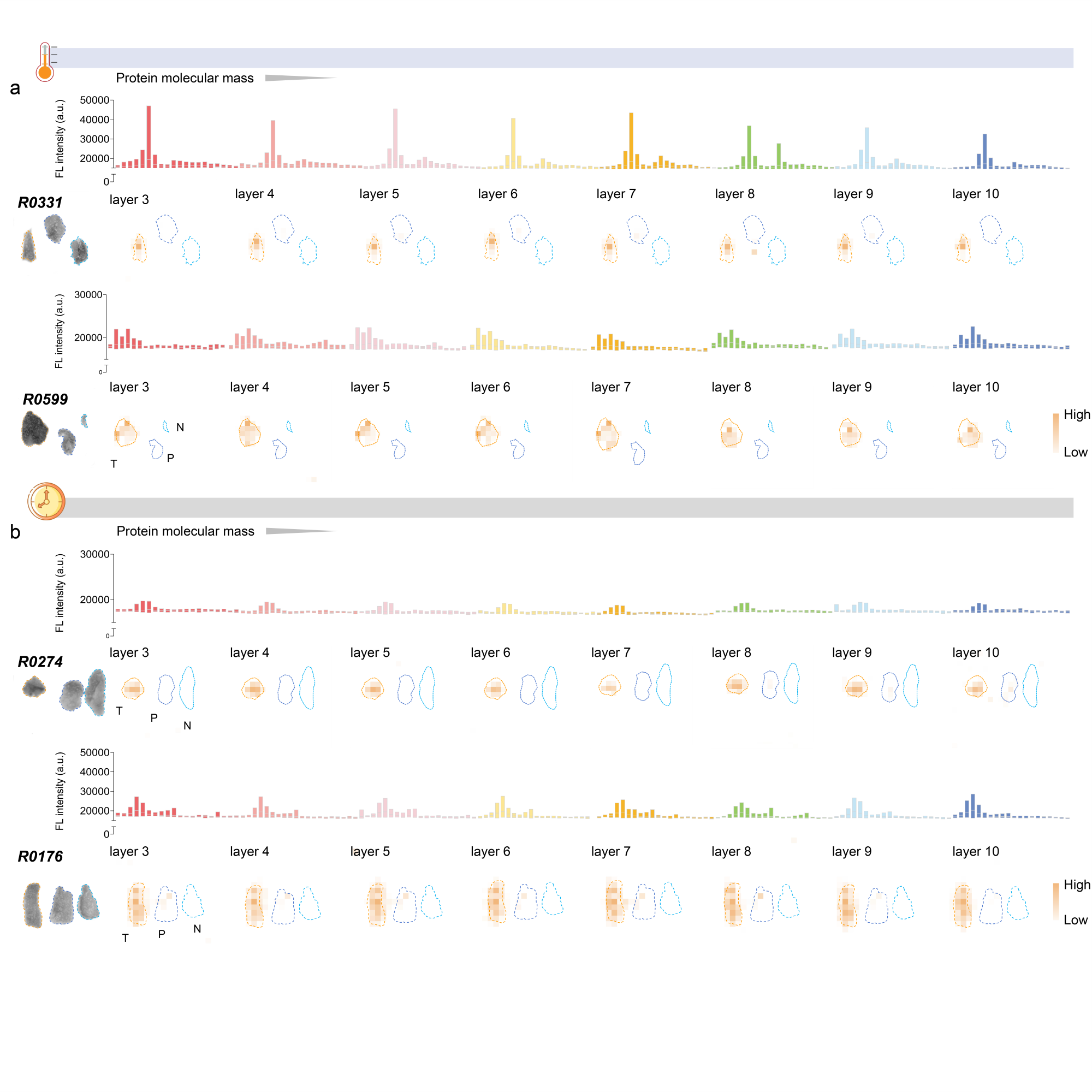
**

**Fig. S21. (a** and **b)** The image of the breast cancer (R0331, R0599, R0274, R0176), Fluorescence images and signal quantification of the eight fractionated layers for the tumor&paracancerous samples following the NIR-FPs-based detection system. (Reaction at 60^o^C for 30 min).

**
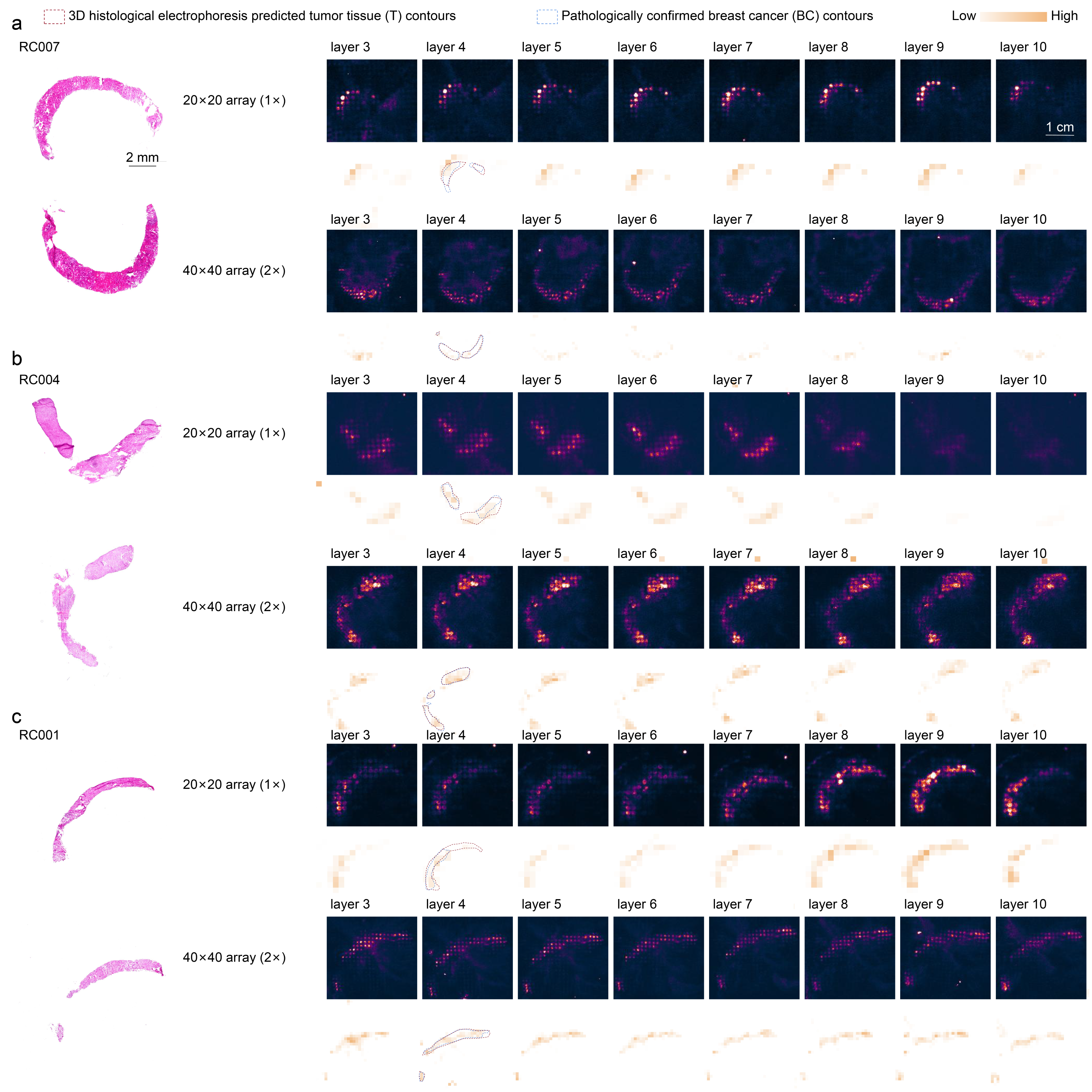
**

**Fig. S22.** H&E staining of breast puncture samples: **(a)** RC007, **(b)** RC004 and **(c)** RC001. Representative fluorescence images and signal quantification of eight separation layers of clinical puncture samples obtained with the NIR-FPs-based detection system under 20×20 and 40×40 microwell array modules. The fluorescence-derived tumor profiles predicted by the NIR-FPs-based detection system were further compared with the corresponding pathologically confirmed tumor profiles.
